# Sub-lethal stress-induced cross-protection against ultraviolet-C in *Salmonella enterica* on raw whole almonds and fresh-cut leafy greens

**DOI:** 10.3389/fmicb.2025.1599380

**Published:** 2025-06-18

**Authors:** Zhao Chen, Jie Zheng, Shirley A. Micallef, Jianghong Meng

**Affiliations:** ^1^Joint Institute for Food Safety and Applied Nutrition, University of Maryland, College Park, MD, United States; ^2^Center for Food Safety and Security Systems, University of Maryland, College Park, MD, United States; ^3^Human Foods Program, United States Food and Drug Administration, College Park, MD, United States; ^4^Department of Plant Science and Landscape, University of Maryland, College Park, MD, United States; ^5^Department of Nutrition and Food Science, University of Maryland, College Park, MD, United States

**Keywords:** *Salmonella enterica*, UV-C, almond, leafy green, sub-lethal stress, cross-protection, *rpoS*, surrogate

## Abstract

Pre-exposure to sub-lethal stress can increase the resistance of foodborne pathogens to inactivation processes, posing potential risks to food safety. This study examined how sub-lethal stress influences the resistance of *Salmonella enterica* to ultraviolet-C (UV-C) treatments on raw whole almonds (RWAs) and fresh-cut leafy greens (FCLGs), investigated the role of *rpoS* in stress-induced cross-protection, and evaluated *Enterococcus faecium* NRRL B-2354 as a surrogate for *S. enterica*. Additionally, we assessed the survival of sub-lethally stressed cells on FCLGs under cold or temperature abuse condition post-UV-C treatment. A cocktail of three *S. enterica* strains, along with *S.* Typhimurium ATCC 14028 and its Δ*rpoS* mutant (IB43), were exposed to desiccation stress, heat shock, oxidation stress, or acid stress. Afterward, stressed and unstressed cells were inoculated onto RWAs and FCLGs, and treated with UV-C (500 μW/cm^2^, 60 min). Treated FCLGs were then stored under cold or temperature abuse condition for 7 days. Results showed that acid-stressed *S. enterica* exhibited greater UV-C resistance on RWAs, while oxidation-stressed cells had increased survival on FCLGs (*p* < 0.05). Under temperature abuse, unstressed, oxidation-stressed, or acid-stressed *S. enterica* were inactivated faster, whereas heat-shocked cells persisted until Day 7. Desiccation-stressed cells rebounded temporarily before inactivation by Day 7. IB43 was more susceptible to UV-C (*p* < 0.05) than the wild-type strain and lacked cross-protection from prior sub-lethal stress exposure, confirming the crucial role of *rpoS* in UV-C resistance and stress adaptation. NRRL B-2354 demonstrated comparable or greater survival than *S. enterica*, supporting its use as a suitable surrogate. These findings highlight the influence of sub-lethal stress on UV-C resistance in *S. enterica* and emphasize the importance of including stress-adapted pathogens in challenge studies to improve food safety.

## 1 Introduction

*Salmonella enterica* is a major cause of foodborne illness in the United States, responsible for an estimated 1.2 million illnesses and 450 deaths annually (CDC, 2004). Raw whole almonds (RWAs) have been linked to multiple outbreaks of *S. enterica*, with evidence of its persistence in food processing environments (CDC, 2004). A notable case occurred from October 2000 to July 2001, with 168 infections in the United States and Canada traced to *S.* Enteritidis phage type 30 (PT30) on RWAs (Isaacs et al., 2005). Current decontamination methods for RWAs, such as propylene oxide fumigation or steam treatments, may pose health risks and compromise product quality (Gao et al., 2011; Jimenez et al., 2015). Recent *S. enterica* outbreaks associated with fresh-cut leafy greens (FCLGs) underscore the urgent need for effective pathogen reduction strategies in this commodity (Herman et al., 2015; CDC, 2024). Chlorine is widely used for FCLG sanitization, but its effectiveness diminishes in the presence of organic matter, and the treatment can also result in the formation of carcinogenic byproducts (López-Gálvez et al., 2010).

Ultraviolet-C (UV-C) irradiation, approved by the United States Food and Drug Administration (FDA, 2000) for microbial control on food surfaces, offers a promising non-thermal alternative postharvest practice. Although not yet currently adopted as standard practice at commercial scale, UV-C has shown promise as a non-thermal decontamination strategy. Studies have demonstrated the potential of UV-C to reduce pathogens on various food products (Ge et al., 2013; Gunter-Ward et al., 2018; Calle et al., 2021). Its application to RWAs and FCLGs has also been explored in prior studies as an alternative to current methods, making it a relevant candidate for future implementation in these food sectors. Ruiz-Hernández et al. (2021) reported a 2.4-log reduction in *S.* Typhimurium on RWAs after 30 min of UV-C treatment, while Escalona et al. (2010) observed reductions between 2.5 and 5.0 logs for *S.* Enteritidis on baby spinach treated with UV-C doses from 2.4 to 24 kJ/m^2^.

*S. enterica* can adapt to various sub-lethal stresses, such as drying, chlorination, heating, and acidification, often encountered during food processing (Wesche et al., 2009; Derossi et al., 2011; Chen and Meng, 2021). These adaptations may provide cross-protection to other stresses, enhancing resistance to subsequent lethal treatments (Capozzi et al., 2009). Alternative sigma factor σ^s^ (RpoS) is crucial for managing stress responses in bacteria (Foster and Spector, 1995). Prior exposure to sub-lethal stress can impair control measures during postharvest handling, potentially increasing pathogen persistence and virulence (Carey et al., 2009). Therefore, understanding the physiological state of a pathogen is essential for accurate sanitation evaluations (Samelis and Sofos, 2002). To simulate real-world conditions, challenge studies should employ cells exposed to similar stresses as those in food processing (National Advisory Committee on Microbiological Criteria for Foods, 2010). Studies have shown that prior exposure to stresses such as desiccation stress, heat shock, or acid stress can elevate UV-C resistance in *S. enterica* on certain food matrices, including coconut liquid endosperm (Gabriel, 2015; Estilo and Gabriel, 2017). However, limited information exists on how such sub-lethal stresses affect pathogen resistance in low-moisture foods and FCLGs.

Moreover, understanding how sub-lethally stressed pathogens survive during post-treatment storage is vital for verifying safe storage conditions. Temperature control is a fundamental aspect of microbial hazard prevention, as mandated by the Food Safety Modernization Act (FSMA; FDA, 2014) and the Food Code 2017, which requires Time/Temperature Control for Safety (TCS) foods like FCLGs to be stored at or below 5°C (FDA, 2017). However, temperature abuse during storage can still occur, enhancing pathogen persistence (Ndraha et al., 2018). Prior research has indicated that the impact of sub-lethal stress on *S. enterica* survival varies with the type of stress and storage conditions (Chen and Meng, 2021).

Non-pathogenic surrogates are essential tools for predicting pathogen behavior in food safety validation studies (Hu and Gurtler, 2017). *Enterococcus faecium* NRRL B-2354 (also known as ATCC 8459) has been recognized as an appropriate surrogate for *Salmonella enterica* in thermal processing of RWAs (Almond Board of California, 2014). Due to its non-pathogenic nature, NRRL B-2354 can be safely used in pilot-scale and industrial settings where handling *S. enterica* would pose safety concerns (Kopit et al., 2014). However, while its effectiveness has been demonstrated under thermal conditions, its behavior under non-thermal treatments such as UV-C exposure—especially when sub-lethally stressed—remains poorly characterized.

**Figure 1:**
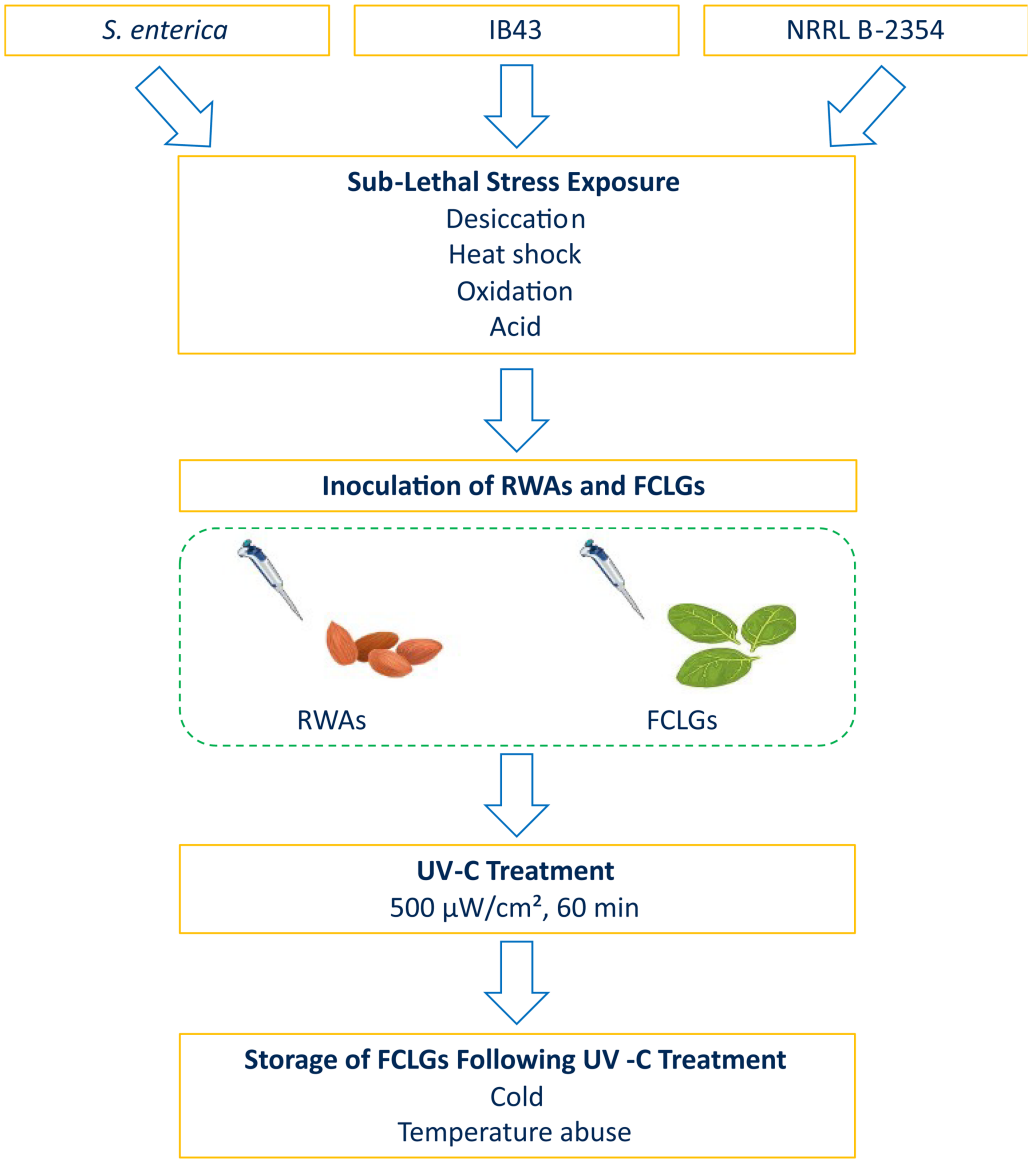
Schematic overview of the experimental procedure. A cocktail of *Salmonella enterica* strains was subjected to sub-lethal stress (desiccation stress, heat shock, oxidation stress, or acid stress), followed by inoculation onto raw whole almonds (RWAs) and fresh-cut leafy greens (FCLGs). Samples were then exposed to ultraviolet-C (UV-C) treatment at 500 μW/cm^2^ for either 30 or 60 min. Post-treatment, FCLGs were stored under cold (4°C) or temperature abuse (35°C for 2 h, then 4°C) condition for 7 days. Bacterial populations were enumerated at multiple time points to assess UV-C resistance and survival dynamics. A Δ*rpoS* mutant (IB43) and *Enterococcus faecium* NRRL B-2354 were also included to evaluate the role of *rpoS* in stress adaptation and the surrogate potential of NRRL B-2354.

To address these knowledge gaps, this study aimed to assess the influence of sub-lethal stress on UV-C resistance in *S. enterica* on RWAs and FCLGs, evaluate the role of *rpoS* in stress-induced cross-protection, and determine the suitability of NRRL B-2354 as a surrogate for *S. enterica*. We also examined the survival of sub-lethally stressed cells on FCLGs under cold or temperature abuse condition. An overview of the experimental design is provided in Figure 1. To our knowledge, this is the first study to systematically evaluate sub-lethal stress-induced cross-protection to UV-C in *S. enterica* on both RWAs and FCLGs.

## 2 Materials and methods

### 2.1 Preparation of bacterial strains

A cocktail of three *S. enterica* strains, including *S.* Enteritidis ATCC BAA-1045 (PT30), *S.* Newport ATCC 6962, and *S.* Typhimurium ATCC 14028, were used to inoculate RWAs. PT30 was chosen due to its association with a RWAs-related outbreak in Canada during 2000–2001 (Isaacs et al., 2005). ATCC 6962 and ATCC 14028 were selected based on the frequent isolation of these two serotypes from RWAs (Bansal et al., 2010). For FCLGs, a mixture of *S.* Enteritidis IEH 399657-02 from organic spinach, *S.* Montevideo 36099 from iceberg lettuce, and *S.* Typhimurium 368477 from Tango lettuce were used. The Δ*rpoS* mutant (IB43), derived from ATCC 14028, was included to investigate the role of *rpoS* in UV-C resistance and stress adaptation. NRRL B-2354 was also evaluated as a surrogate for sub-lethally stressed *S. enterica*. All strains were rendered resistant to 100 μg/mL rifampicin using the gradient plate method (Smith et al., 1982), which involved spreading bacterial cultures onto tryptic soy agar (TSA; Fisher Scientific Inc., Hampton, NH, United States) containing a gradually increasing concentration of rifampicin across the plate to select for resistant mutants. To ensure that rifampicin resistance (Rif^R^) did not impact stress responses, multiple resistant strains were isolated and compared to the wild-type strain under identical conditions. Only strains exhibiting no significant differences in growth or stress tolerance were selected for this study. Stock cultures of resistant strains were stored at −80°C in tryptic soy broth (TSB; Fisher Scientific Inc.) containing 25% glycerol until further use.

### 2.2 Preparation of sub-lethally stressed cells

Each strain was streaked from stock cultures and grown overnight at 35°C on TSA. A single colony was transferred to TSB, followed by two successive overnight incubations at 35°C. Cells were then harvested and washed with 0.85% saline containing 0.5% Tween-80, a non-ionic surfactant used to reduce cell aggregation and ensure even dispersion (Brandl and Huynh, 2014). The cell suspension was adjusted to 9.0 log CFU/mL, corresponding to an optical density of 0.7 at 600 nm, as confirmed by plate counts. Equal volumes of the three *S. enterica* strains were combined to prepare a mixed-strain inoculum prior to exposure to sub-lethal stress.

Bacterial cells were subjected to sub-lethal desiccation stress, heat shock, oxidation stress, or acid stress (Dhakal et al., 2019; Estilo and Gabriel, 2017; Koutsoumanis and Sofos, 2004; Singh et al., 2010). The required time of exposure to each sub-lethal stress, which stresses the cells the most without causing lethality, was determined based on the method outlined by Dhakal et al. (2019). Briefly, bacterial cells (9.0 log CFU/mL) were suspended in: (1) Desiccation stress: 1 mL of 1 M NaCl (a_w_ = 0.96) and incubated at 22°C for 2 h, (2) Oxidation stress: 1 mL of TSB, mixed with 1 mL of 300 mg/L sodium hypochlorite (final concentration = 150 mg/L), and incubated at 22°C for 2 h, (3) Heat shock: 1 mL of TSB and incubated at 48°C for 60 min, or (4) Acid stress: 1 mL of TSB adjusted to pH 5.0 with 1 M hydrochloric acid and incubated at 30°C for 1.5 h. Unstressed cells in sterile saline containing 0.5% Tween-80 served as the unstressed control.

### 2.3 Inoculation of RWAs and FCLGs

Conventionally grown RWAs were sourced from a commercial grower in Earlimart, CA, United States, and sorted to eliminate damaged or blemished kernels prior to experiments. Each sample unit included ten RWAs of uniform size. Fresh-cut conventionally grown leafy greens, including baby spinach, baby tango lettuce, and radicchio, were purchased from a local grocery store and refrigerated at 4°C until use. Twenty-one leaves (seven leaves for each leafy green) of uniform size, free from visible defects, constituted each sample unit. To ensure precise application of a known number of cells for each sample unit, RWAs and FCLGs of each sample unit were spot inoculated with 100 μL of sub-lethally stressed or unstressed cells (approximately 6.0 log CFU/sample unit), followed by air drying at 22°C for 60 min.

### 2.4 UV-C treatment

UV-C lamps in a UV CLAVE ultraviolet chamber (Benchmark Scientific, Inc., Sayreville, NJ, United States) were warmed up for 15 min. Inoculated samples were placed 15 cm from the lamps, with UV-C irradiance set at 500 μW/cm^2^ for 60 min, and collected at 0, 1, 3, 5, 10, 15, 30, and 60 min. Untreated controls consisted of bacterial cells inoculated on samples and held under the same conditions for 60 min without UV-C exposure.

### 2.5 Storage of FCLGs following UV-C treatment

Following the 30- or 60-min UV-C treatment, FCLGs were stored under two specified temperature conditions for 7 days: (1) constant cold storage at 4°C for the entire duration or (2) temperature abuse, involving exposure to 35°C for 2 h followed by storage at 4°C for the remaining seven-day period (Huang et al., 2019). Sampling occurred on days 0, 1, 2, 4, and 7.

### 2.6 Microbiological analysis

Samples were homogenized with 10 mL sterile 0.85% saline containing 0.5% Tween-80 by hand massaging for 3 min. Decimal serial dilutes were then prepared using sterile 0.85% saline containing 0.5% Tween-80, and 100 μL aliquots were spread in triplicate onto TSA supplemented with 100 μg/mL rifampicin (TSA-R), followed by incubation at 35°C for 24 h. Colonies on each plate were counted, and the average of three counts was recorded and expressed as log CFU/sample unit. Samples negative for *S. enterica* by direct plating were pre-enriched in universal pre-enrichment broth (UPB; Becton, Dickinson and Company, Sparks, MD, United States) at 35°C for 24 h and then enriched in Rappaport-Vassiliadis (RV) broth (Becton, Dickinson and Company) at 42°C for 24 h. Enriched samples were then selectively plated onto xylose lysine desoxycholate (XLD; Fisher Scientific Inc.) and incubated at 35°C for 24 h. The limits of detection for direct plating and enrichment were 1.0 and 0.0 log CFU/sample unit, respectively.

### 2.7 Mathematical modeling

Six non-linear models were employed to simulate survival curves: Weibull, double Weibull, log-linear with tail, log-linear with shoulder and tail, biphasic, and biphasic with shoulder (Cerf and Métro, 1977; Coroller et al., 2006; Geeraerd et al., 2000; Geeraerd et al., 2005; Mafart et al., 2002). The Regression Wizard Module in SigmaPlot 15.0 (Systat Software Inc., San Jose, CA, United States) facilitated the simulation of survival curves using these models. Non-linear regression modeling parameters were set to ensure convergence: iterations = 200, step size = 1, and tolerance = 1 × 10^−10^. The performance of each model was evaluated based on adjusted *R*^2^ and root mean square error (RMSE). Higher adjusted *R*^2^ values approaching 1 and lower RMSE values approaching 0 indicate better simulation of observed data.

### 2.8 Non-metric multidimensional scaling

Non-metric multidimensional scaling (NMDS) based on Bray-Curtis distances was used to visualize multivariate clustering and explore the influence of food matrix, stress, microorganism, and model parameters on microbial inactivation. Analyses were conducted using the vegan 2.6–10 (Oksanen, 2013) and ggplot2 3.5.2 (Wickham, 2011) packages in R 4.5.0. Groupings were defined by: (1) food matrix (RWAs vs. FCLGs), (2) stress (no stress, desiccation stress, heat shock, oxidation stress, or acid stress), (3) microorganism (*S. enterica*, IB43, vs. NRRL B-2354), and (4) model parameters. Permutational multivariate analysis of variance (PERMANOVA) was performed using the adonis2() function from the vegan package in R, with significance set at *α* = 0.05. NMDS stress values were interpreted as follows: < 0.05 (excellent), 0.05–0.10 (good), 0.10–0.20 (fair), and > 0.20 (poor), based on Dexter et al. (2018).

### 2.9 Statistical analysis

All results were obtained from three independent trials. Bacterial counts were expressed as log CFU/sample unit. Statistical differences among treatments were assessed using analysis of variance (ANOVA) followed by the Holm–Šidák post hoc test.

## 3 Results

### 3.1 Survival of sub-lethally stressed *Salmonella enterica* on RWAs and FCLGs

#### 3.1.1 RWAs

**Figure 2:**
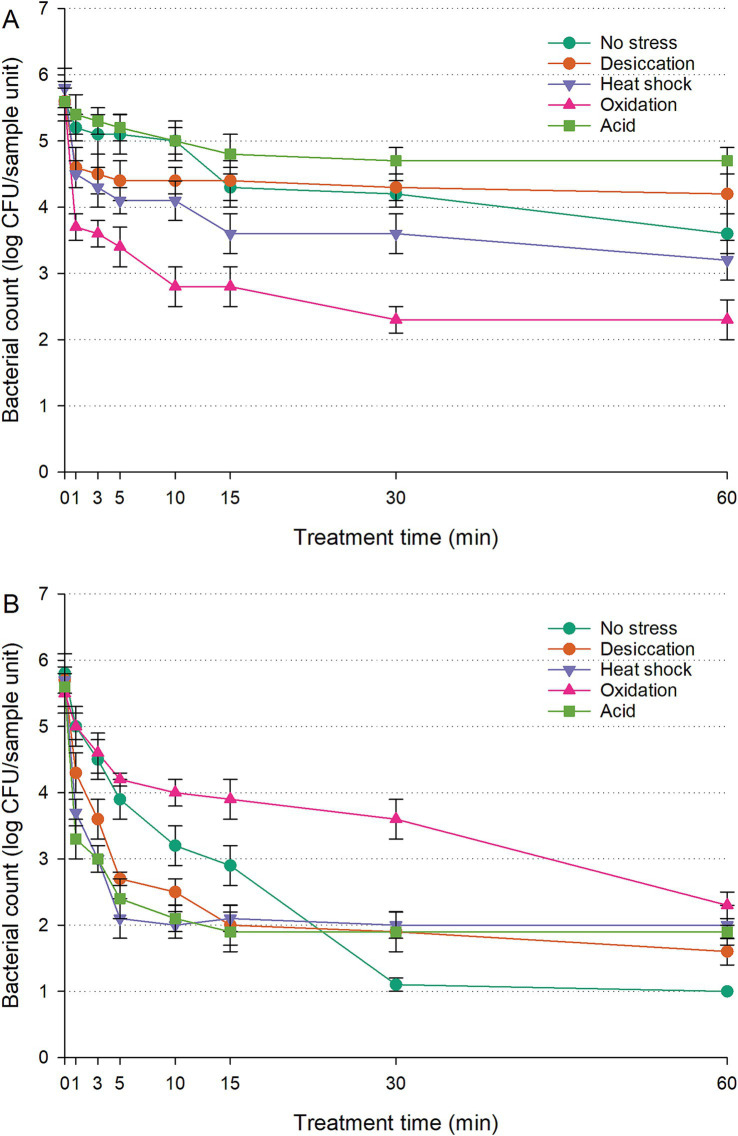
Survival of *Salmonella enterica* on raw whole almonds **(A)** and fresh-cut leafy greens **(B)** during ultraviolet-C treatment, with or without prior exposure to sub-lethal stress. Error bars represent standard deviations from three independent trials. Bacterial counts plotted as 1.0 log CFU/sample unit without error bars were below the limit of detection by direct plating but were detectable by enrichment.

The populations of untreated controls (cells inoculated on RWAs without UV-C exposure) remained stable throughout the 60-min treatment, showing no significant change (*p* > 0.05). The survival curves of *S. enterica* on RWAs under UV-C treatment, with or without prior sub-lethal stress exposure, are shown in Figure 2A; Supplementary Figure S1I. Unstressed cells gradually declined from 6.0 to 5.0 log CFU/sample unit within 10 min, followed by an additional two-log reduction by the end of the treatment. Acid-stressed cells maintained significantly higher counts than unstressed cells throughout UV-C exposure (*p* < 0.05), leveling off around 4.7–4.8 log CFU/sample unit after the initial 10 min. Desiccation-stressed cells initially showed greater sensitivity to UV-C (*p* < 0.05) in the first 10 min but exhibited similar survival rates to unstressed cells between 15 and 30 min. By 60 min, desiccation-stressed cells had significantly higher populations than unstressed cells (*p* < 0.05). Unstressed cells showed better UV-C survival than heat-shocked or oxidation-stressed cells (*p* < 0.05). Oxidation-stressed cells experienced the steepest decline, dropping to 3.7 log CFU/sample unit within 1 min and further decreasing to 2.3 log CFU/sample unit by the end of the treatment. In contrast, heat-shocked cells demonstrated moderate survival, consistently maintaining populations 0.7–1.3 log CFU/sample unit higher than oxidation-stressed cells (*p* < 0.05).

#### 3.1.2 FCLGs

The populations of untreated controls (cells inoculated on FCLGs without UV-C exposure) remained unchanged over the 60-min treatment, with no statistically significant variations (*p* > 0.05). The survival of *S. enterica* on FCLGs during UV-C treatment varied significantly depending on prior sub-lethal stress exposure, showing distinct UV-C resistance patterns (Figure 2B; Supplementary Figure S1II). Unstressed cells declined steadily to 3.2 log CFU/sample unit within the first 10 min. However, oxidation-stressed cells demonstrated greater resistance than other conditions (*p* < 0.05), decreasing more gradually and retaining a population of 4.0 log CFU/sample unit after 10 min. In contrast, desiccation-stressed, heat-shocked, or acid-stressed cells were more sensitive to UV-C than unstressed cells, displaying similar vulnerability patterns (*p* > 0.05). These stress conditions lowered UV-C tolerance, leaving cells more susceptible. While unstressed cells showed higher UV-C resistance within the first 15 min compared to desiccation-stressed, heat-shocked, or acid-stressed cells (*p* < 0.05), their sensitivity increased after 30 min of exposure (*p* < 0.05).

### 3.2 Survival of sub-lethally stressed IB43 on RWAs and FCLGs

#### 3.2.1 RWAs

**Figure 3:**
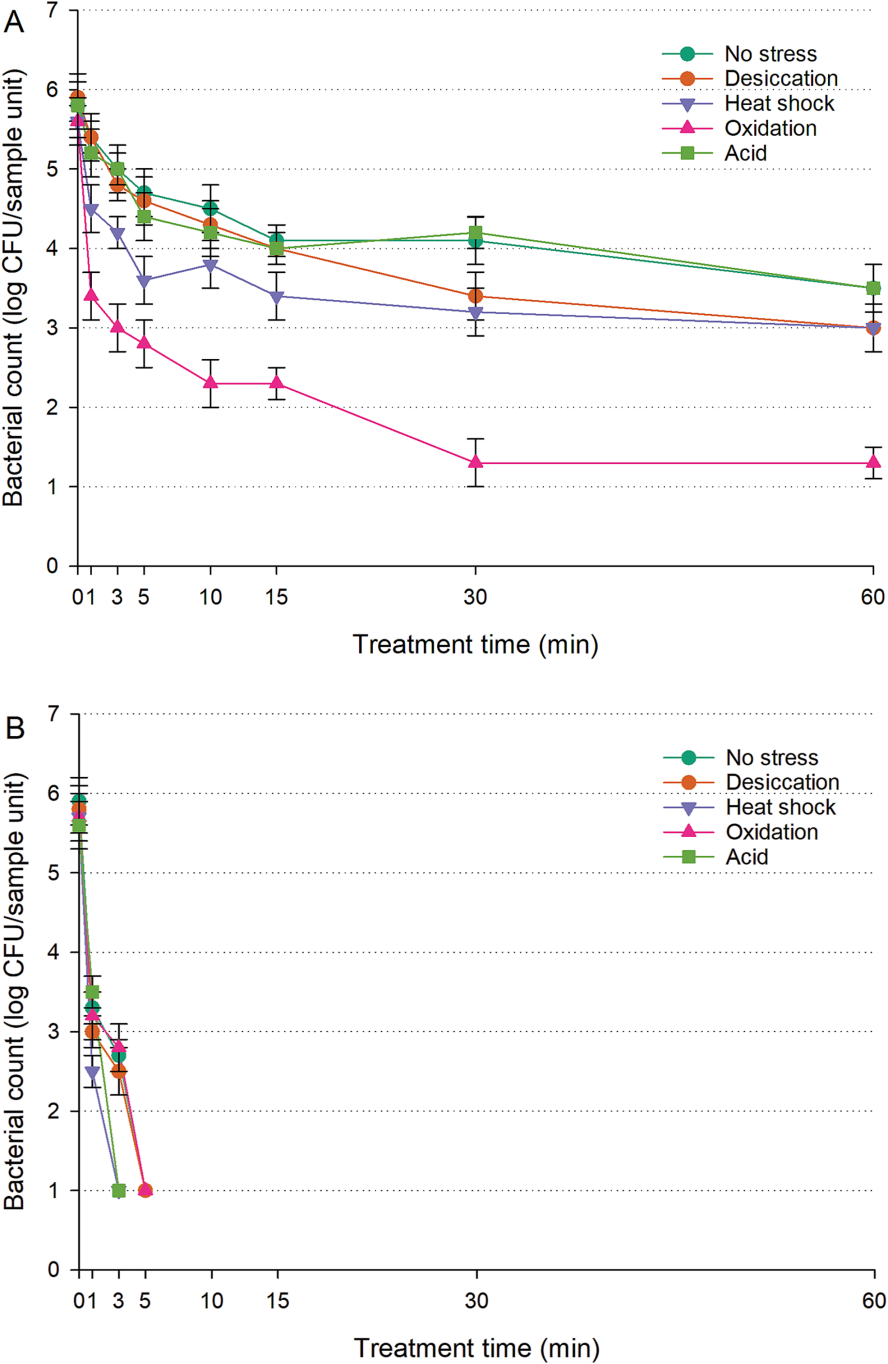
Survival of *Salmonella* Typhimurium IB43 (Δ*rpoS* mutant of *S.* Typhimurium ATCC 14028 wild-type) on raw whole almonds **(A)** and fresh-cut leafy greens **(B)** during ultraviolet-C treatment, with or without prior exposure to sub-lethal stress. ‘Error bars represent standard deviations from three independent trials. Bacterial counts plotted as 1.0 log CFU/sample unit without error bars were below the limit of detection by direct plating but were detectable by enrichment. Bacterial counts are not shown at certain time points because no cells were detected, even after enrichment.

The populations of untreated controls (cells inoculated on RWAs without UV-C exposure) exhibited no significant fluctuations over the 60-min treatment (*p* > 0.05). The wild-type data were excluded from Figure 3 to enhance clarity and eliminate redundancy; however, they were provided in Supplementary Figure S2 for reference. The lack of a functional RpoS system diminished survival in stressed IB43 cells compared to the wild-type (Figure 3A; Supplementary Figures S1I, S2A) (*p* < 0.05). However, no notable difference was observed between unstressed IB43 and wild-type cells under UV-C exposure on RWAs (*p* > 0.05). Among the stressed cells, oxidation-stressed IB43 declined most rapidly, reaching 1.3 log CFU/sample unit within 30 min—significantly more than other groups (*p* < 0.05). Within the initial 15 min, no significant difference was seen between unstressed and desiccation-stressed IB43 (*p* > 0.05), though unstressed cells showed better survival afterward. Heat-shocked IB43 populations remained consistently lower than unstressed cells (*p* < 0.05), while acid-stressed IB43 exhibited similar survival to unstressed cells (*p* > 0.05) throughout the treatment.

#### 3.2.2 FCLGs

Untreated controls (cells inoculated on FCLGs without UV-C exposure) maintained a consistent population over the 60-min period, with no significant changes detected (*p* > 0.05). On FCLGs, the population of unstressed IB43 rapidly declined to undetectable levels by enrichment within the first 10 min of UV-C exposure (Figure 3B), whereas unstressed wild-type remained detectable by direct plating throughout the entire exposure period (Supplementary Figures S1II, S2B). This rapid reduction was consistent across all sub-lethal stresses assessed. Stressed IB43 cells were eliminated within 10 min, with heat-shocked IB43 showing the sharpest decline, reaching undetectable levels by enrichment within just 5 min (*p* < 0.05). Desiccation- or oxidation-stressed IB43 exhibited survival patterns similar to unstressed cells throughout the UV-C exposure, while both unstressed and acid-stressed IB43 showed no significant differences in the first minute (*p* > 0.05). However, unstressed cells demonstrated better survival after 3 min of UV-C exposure.

### 3.3 Survival of sub-lethally stressed NRRL B-2354 on RWAs and FCLGs

#### 3.3.1 RWAs

**Figure 4:**
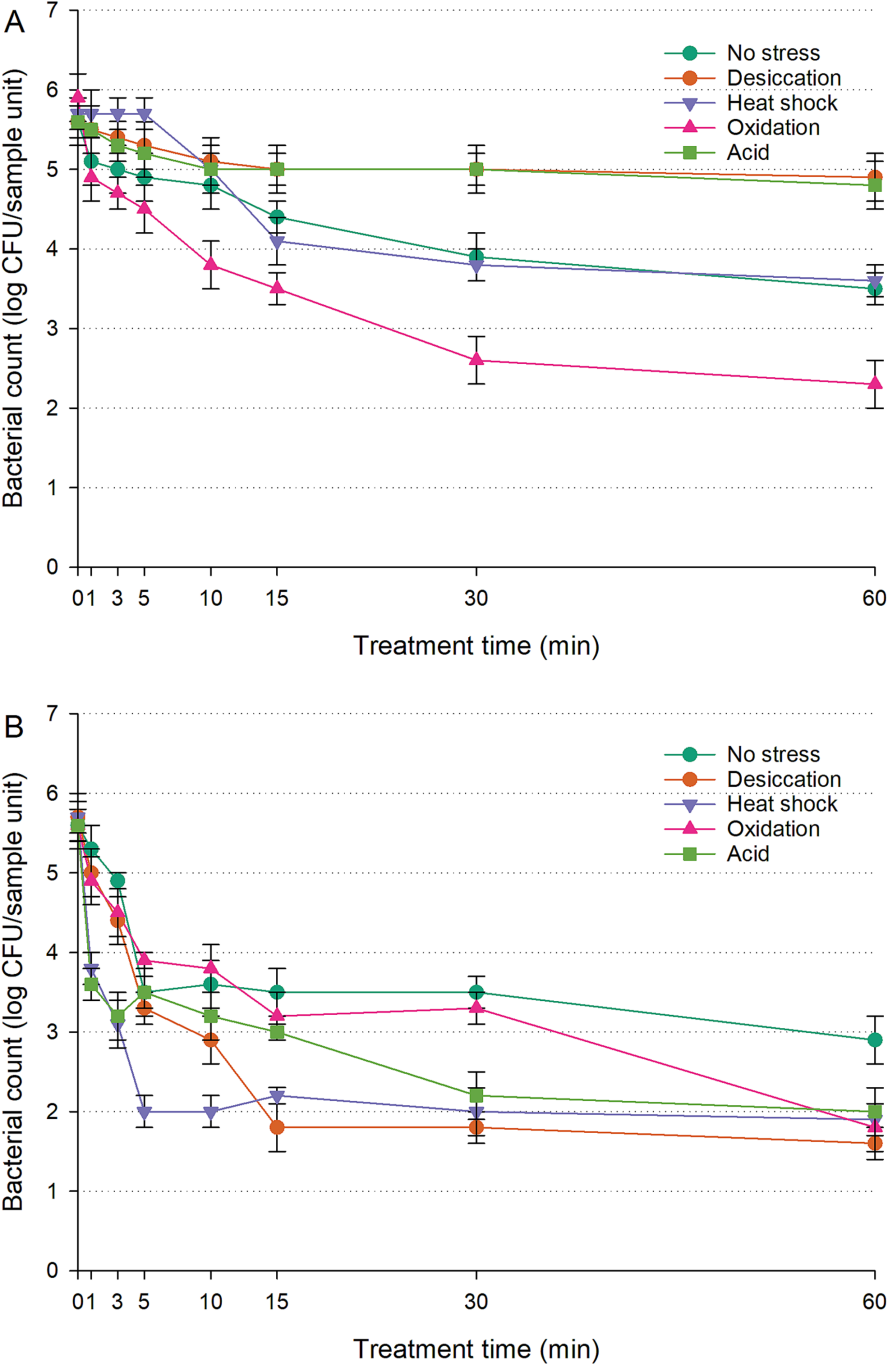
Survival of *Enterococcus faecium* NRRL B-2354 on raw whole almonds **(A)** and fresh-cut leafy greens **(B)** during ultraviolet-C treatment, with or without prior exposure to sub-lethal stress. Error bars represent standard deviations from three independent trials.

The populations of untreated controls (cells inoculated on RWAs without UV-C exposure) remained constant throughout the 60-min period, showing no significant differences (*p* > 0.05). Both unstressed and acid-stressed NRRL B-2354 exhibited similar UV-C resistance to *S. enterica* (*p* > 0.05) (Figure 4A; Supplementary Figure S1I). However, NRRL B-2354 showed significantly higher resistance than *S. enterica* after exposure to desiccation stress, oxidation stress, or heat shock (*p* < 0.05). The respective population differences were 0.6–0.9, 0.3–1.2, and 0.4–1.6 log CFU/sample unit. Notably, only a slight reduction (0.7–0.8 log CFU/sample unit) was observed for desiccation- or acid-stressed NRRL B-2354, further highlighting its resistance.

#### 3.3.2 FCLGs

Untreated controls (cells inoculated on FCLGs without UV-C exposure) showed no measurable changes in population over the 60-min period (*p* > 0.05). NRRL B-2354 demonstrated greater UV-C resistance than *S. enterica* under unstressed conditions, as well as after desiccation or acid stress (*p* < 0.05) (Figure 4B; Supplementary Figure S1II). The population differences ranged from 0.3 to 2.4 log CFU/sample unit under no stress, 0.4 to 0.8 log CFU/sample unit following desiccation stress, and 0.1 to 1.1 log CFU/sample unit after acid stress. However, NRRL B-2354 and *S. enterica* exhibited similar survival patterns following heat shock or oxidation stress (*p* > 0.05).

### 3.4 Mathematical modeling

Modeling of bacterial inactivation demonstrated non-linear survival curves with pronounced tailing, suggesting the presence of phenotypic heterogeneity or persister cells (Supplementary Results and Discussion; Supplementary Tables S1–S6). Among all models, double Weibull provided the best fit for most datasets, supporting its value in describing complex inactivation kinetics (Supplementary Table S2).

### 3.5 Multivariate analysis of inactivation patterns

**Figure 5:**
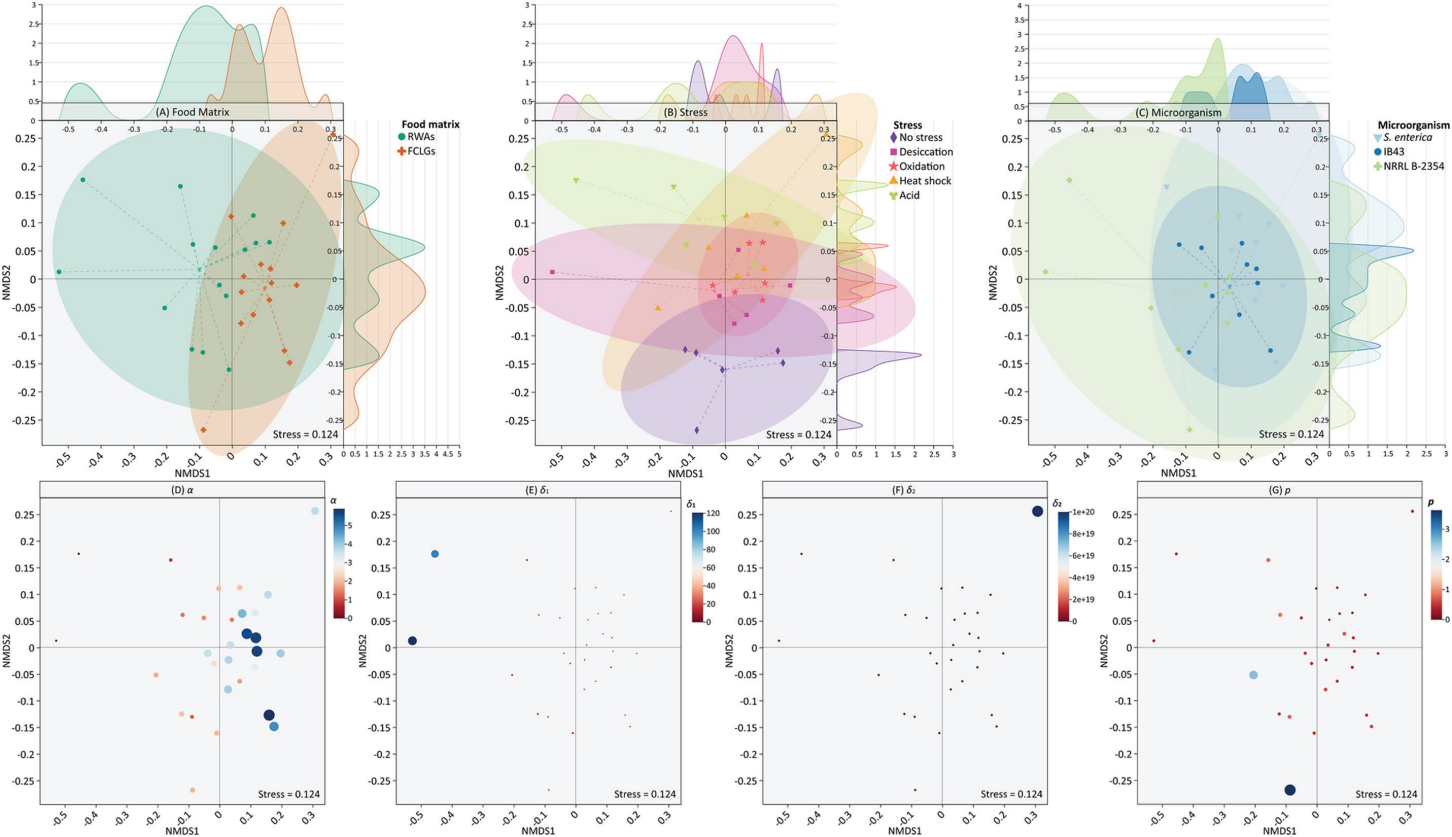
Non-metric multidimensional scaling (NMDS) based on grouping factors including food matrix **(A)**, stress **(B)**, microorganism **(C)**, and model parameters [*α* **(D)**, *δ*_1_ **(E)**, *δ*_2_ **(F)**, and *p* **(G)**] for the survival of *Salmonella enterica*, *S.* Typhimurium IB43, and *Enterococcus faecium* NRRL B-2354 on raw whole almonds (RWAs) and fresh-cut leafy greens (FCLGs) during ultraviolet-C treatment, with or without prior exposure to sub-lethal stress. Model parameters, including *α* [difference between the sensitive subpopulation and the resistant subpopulation (log CFU/sample unit)], *δ*_1_ [time of the first decimal reduction of the sensitive subpopulation (min)], *δ*_2_ [time of the first decimal reduction of the resistant subpopulation (min)], and *p* (shape factor), were derived from the double Weibull model but their data points were highly dispersed, precluding the use of representative confidence ellipses. Ellipses represent groupings at a 95% confidence level. Top and right density plots show the distribution of data points along NMDS1 and NMDS2 axes, respectively. The size of each data point for model parameters is proportional to the magnitude of its value.

NMDS plots (Figure 5) visualize the multivariate distribution of survival curve data across food matrix, stress, microorganism, and model parameter groupings. Double Weibull parameters, including *α* [difference between the sensitive subpopulation and the resistant subpopulation (log CFU/sample unit)], *δ*_1_ [time of the first decimal reduction of the sensitive subpopulation (min)], *δ*_2_ [time of the first decimal reduction of the resistant subpopulation (min)], and *p* (shape factor), were used for NMDS due to their overall good performance in capturing the non-linear survival behavior observed across conditions (Supplementary Table S2). The data points of model parameters were highly dispersed, making it impossible to define clear clusters or representative confidence ellipses.

To assess the multivariate structure of survival curve parameters across treatments, a NMDS ordination was performed followed by PERMANOVA (Table 1). While no statistically significant differences were detected (*p* > 0.05), moderate effect sizes for parameters such as *α* (*R* = 0.206) and *δ*_2_ (*R* = 0.261) suggested trends in curve shape potentially influenced by stress or microorganism. Although the multivariate analysis did not reveal significant clustering, the observed separation trends in *α* and *δ*_2_ parameters demonstrated potential variation in microbial inactivation kinetics related to physiological stress adaptations.

**Table 1:** Permutational multivariate analysis of variance based on non-metric multidimensional scaling, assessing centroid differences among food matrix, microorganism, stress, and model parameters for each grouping factor for the inactivation of *Salmonella enterica*, *S.* Typhimurium IB43, and *Enterococcus faecium* NRRL B-2354 on raw whole almonds (RWAs) and fresh-cut leafy greens (FCLGs) under ultraviolet-C treatment, with or without prior exposure to sub-lethal desiccation stress, heat shock, oxidation stress, or acid stress.

Factor^a^	*R*	*P*
Food matrix	0.014	0.282
Stress	0.002	0.403
Microorganism	0.021	0.258
*α*	0.206	0.130
*δ*_1_	−0.204	0.851
*δ*_2_	0.261	0.163
*p*	0.051	0.332

a Food matrix: RWAs vs. FCLGs; microorganism: *S. enterica*, *S.* Typhimurium IB43, vs. NRRL B-2354; stress: no stress, desiccation stress, heat shock, oxidation stress, or acid stress; model parameters, including *α* [difference between the sensitive subpopulation and the resistant subpopulation (log CFU/sample unit)], *δ*_1_ [time of the first decimal reduction of the sensitive subpopulation (min)], *δ*_2_ [time of the first decimal reduction of the resistant subpopulation (min)], and *p* (shape factor), were derived from the double Weibull model.

### 3.6 Survival of sub-lethally stressed *Salmonella enterica*, IB43, and NRRL B-2354 on FCLGs under cold or temperature abuse condition after UV-C exposure

#### 3.6.1 Salmonella enterica

**Figure 6:**
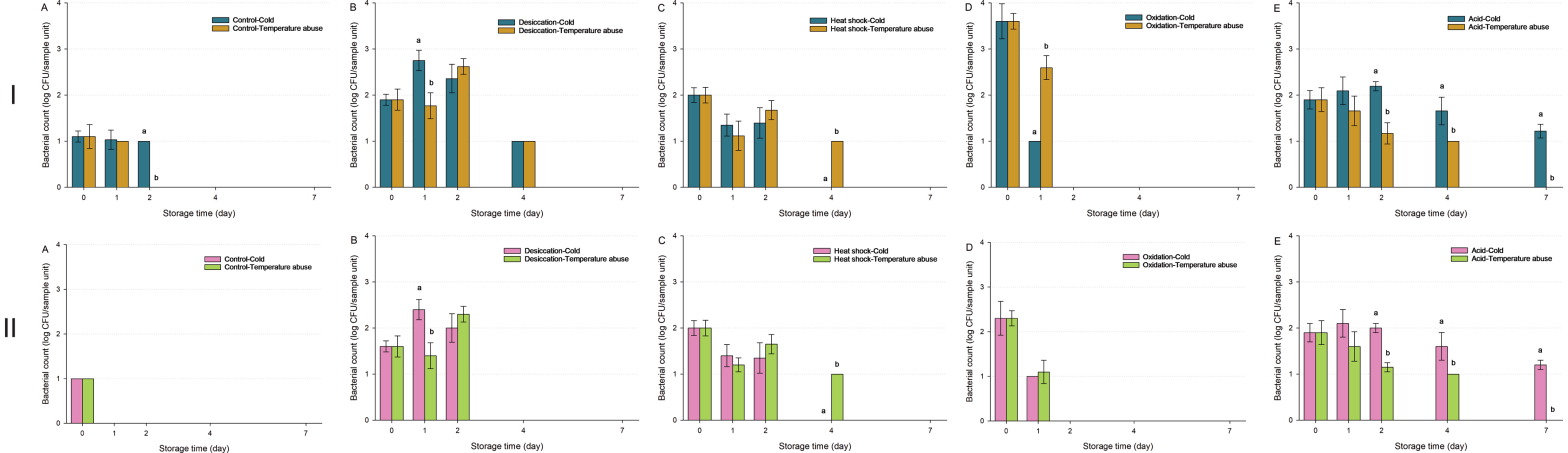
Survival of *Salmonella enterica* on fresh-cut leafy greens during a seven-day storage period under cold or temperature abuse condition following ultraviolet-C (UV-C) treatment. Bacterial survival is shown for unstressed cells **(A)** and cells subjected to sub-lethal desiccation stress **(B)**, heat shock **(C)**, oxidation stress **(D)**, or acid stress **(E)**. UV-C treatment was applied for 30 **(I)** or 60 min **(II)**. Error bars represent standard deviations from three independent trials. Bacterial counts plotted as 1.0 log CFU/sample unit without error bars were below the limit of detection by direct plating but were detectable by enrichment. Bacterial counts are not shown at certain time points because no cells were detected, even after enrichment. Different letters above bars indicate significant differences (*p* < 0.05) between cold and temperature abuse conditions.

Following a 30-min UV-C treatment (Figure 6I), reductions in unstressed and acid-stressed *S. enterica* were significantly accelerated under temperature abuse (*p* < 0.05). In contrast, heat-shocked cells exhibited greater persistence under temperature abuse (*p* < 0.05), with viable populations detectable until Day 7. Oxidation-stressed cells showed higher populations under temperature abuse compared to cold storage (*p* < 0.05); however, all were undetectable by Day 2. Desiccation-stressed cells exhibited temporary population rebounds on Day 1 (cold) or Day 2 (temperature abuse) before declining to undetectable levels by Day 7.

For the 60-min UV-C treatment (Figure 6II), similar trends were observed across stress conditions, but initial bacterial populations were lower due to the longer exposure, leading to faster inactivation for unstressed and desiccation-stressed *S. enterica*. Notably, extended UV-C exposure diminished the effect of storage temperature, as oxidation-stressed cells treated for 60 min showed no significant difference in survival between cold and temperature abuse conditions (*p* > 0.05).

#### 3.6.2 IB43

**Figure 7:**
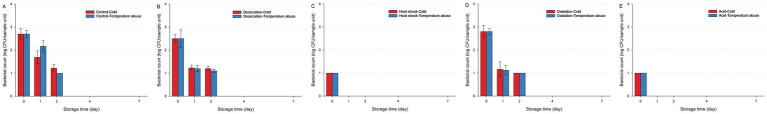
Survival of *Salmonella* Typhimurium IB43 (the Δ*rpoS* mutant of *S.* Typhimurium ATCC 14028) on fresh-cut leafy greens during a seven-day storage period under cold or temperature abuse condition following ultraviolet-C (UV-C) treatment. Bacterial survival is shown for unstressed cells **(A)** and cells subjected to sub-lethal desiccation stress **(B)**, heat shock **(C)**, oxidation stress **(D)**, or acid stress **(E)**. UV-C treatment was applied for 30 min. Error bars represent standard deviations from three independent trials. Bacterial counts plotted as 1.0 log CFU/sample unit without error bars were below the limit of detection by direct plating but were detectable by enrichment. Bacterial counts are not shown at certain time points because no cells were detected, even after enrichment.

For IB43, exposure to a 30-min UV-C treatment resulted in complete inactivation by Day 4 under both cold and temperature abuse conditions, irrespective of prior stress (Figure 7). Heat-shocked or acid-stressed IB43 cells were especially vulnerable (*p* < 0.05), with no survivors by Day 1 in either environment. Unstressed, desiccation-stressed, or oxidation-stressed cells declined slightly more slowly under temperature abuse, though all were inactivated by Day 4. In contrast, the wild-type displayed adaptive responses, where temperature abuse allowed temporary recovery in desiccation-stressed or heat-shocked cells (Supplementary Figure S3).

#### 3.6.3 NRRL b-2354

**Figure 8:**
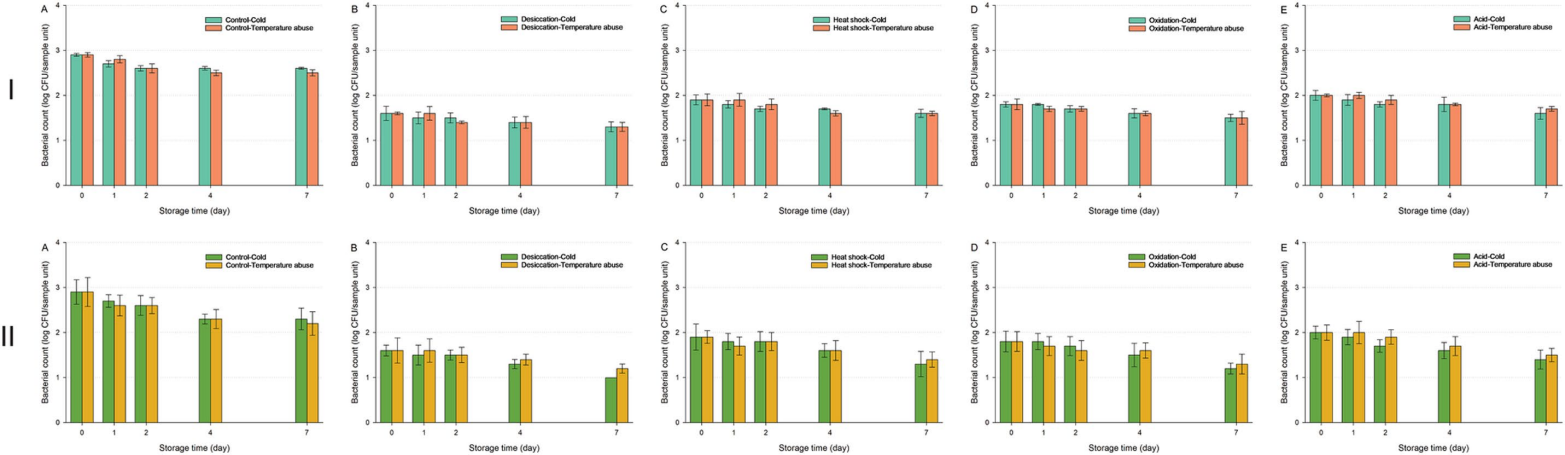
Survival of *Enterococcus faecium* NRRL B-2354 on fresh-cut leafy greens during a seven-day storage period under cold or temperature abuse condition following ultraviolet-C (UV-C) treatment. Bacterial survival is shown for unstressed cells **(A)** and cells subjected to sub-lethal desiccation stress **(B)**, heat shock **(C)**, oxidation stress **(D)**, or acid stress **(E)**. UV-C treatment was applied for 30 **(I)** or 60 min **(II)**. Error bars represent standard deviations from three independent trials. Bacterial counts plotted as 1.0 log CFU/sample unit without error bars were below the limit of detection by direct plating but were detectable by enrichment. Bacterial counts are not shown at certain time points because no cells were detected, even after enrichment.

NRRL B-2354 populations remained stable across cold and temperature abuse conditions, showing only minor reductions by Day 7 across all sub-lethal stresses (Figure 8). Similar survival patterns across both storage conditions indicated minimal variation in bacterial count reduction.

## 4 Discussion

This study provides novel insights into how sub-lethal stress influences UV-C resistance in *S. enterica* on RWAs and FCLGs. By evaluating the role of the general stress response regulator *rpoS* and comparing *S. enterica* to the non-pathogenic surrogate NRRL B-2354, we expanded our current understanding of cross-protection mechanisms and microbial survival under UV-C treatment.

Prior research has shown that exposure to sub-lethal conditions can induce cross-protection against subsequent stresses. Gabriel (2015) reported increased *D*-values for desiccation- (a_w_ = 0.85, 4–24 h) or acid- (pH 4.5, 18–24 h) stressed *S.* Enteritidis, Infantis, and Montevideo in coconut liquid endosperm compared to unstressed cells. Similarly, Mutz et al. (2020) observed a 14-fold increase in UV-C dose requirements for the first log reduction of *S.* Typhimurium in dry-fermented sausage (a_w_ = 0.85, pH 5.4) after a 24-h habituation period. Our findings supported these observations: desiccation- or acid-stressed *S. enterica* exhibited improved survival on RWAs during UV-C exposure, likely due to the upregulation of protective stress response systems. These results are particularly relevant because mild acidification and drying are common food processing steps, which could unintentionally prime pathogens for increased resistance during sanitation. Our modeling data reinforces the need to consider subpopulations with elevated resistance when evaluating disinfection efficacy, as these cells can disproportionately influence survival outcomes and pose persistent risks.

In contrast, oxidation stress caused the greatest UV-C sensitivity in *S. enterica*, especially on RWAs. This aligns with previous findings showing that chlorine-induced oxidative damage impairs DNA repair and cellular function (Chaves et al., 2019). Oxidation stress may compromise membrane integrity in a way that interacts specifically with the low-moisture RWA surface—possibly intensifying UV-C-induced damage (Fukuzaki, 2006). In contrast, certain properties of FCLGs (e.g., residual moisture, antioxidant compounds, or leaf surface chemistry) might help buffer oxidative damage or support limited recovery during UV-C exposure. Importantly, other stressed cells did not show the same pattern, indicating that the interaction between oxidation stress and food surface may be uniquely synergistic. The outcome likely reflects a complex interplay between the physiological state of the cells and food surface characteristics such as moisture availability, matrix composition, and UV-C reflectivity or absorption. Importantly, since chlorine is a widely used disinfectant in produce processing (Goodburn and Wallace, 2013), our data suggests that oxidation-stressed cells may be more vulnerable to UV-C, offering a potential advantage for sequential disinfection strategies.

However, on FCLGs, oxidation-stressed cells displayed greater survival during UV-C exposure than on RWAs, highlighting the matrix-dependent nature of bacterial survival. This variation likely stems from differences in surface texture and hydrophobicity (Fan et al., 2017). Rough or irregular RWA surfaces can shield bacteria from UV-C exposure by creating micro-shadows, while smoother FCLG surfaces may allow more direct UV-C irradiation. Adhikari et al. (2015) observed that UV-C was more effective in killing *Escherichia coli* O157:H7 and *Listeria monocytogenes* on smoother fruit surfaces (e.g., apples and pears) than on rougher ones (e.g., cantaloupes and strawberries). Similarly, Mukhopadhyay et al. (2014) found lower reductions of *S. enterica* and *E. coli* O157:H7 on tomato stem scars compared to smoother areas. To support this notion, scanning electron microscopy by Yun et al. (2013) revealed that UV-C struggles to reach bacteria nestled within surface irregularities on plants, with these structural features forming protective niches that shield pathogens from UV-C exposure. Interestingly, we observed that oxidation-stressed cells were more susceptible on RWAs but more resistant on FCLGs, reinforcing that UV-C effectiveness is highly dependent on the food matrix. These findings suggest that UV-C-based interventions must be tailored to the surface characteristics of the specific commodity.

Our study also emphasizes the critical role of *rpoS* in protection against UV-C exposure. The Δ*rpoS* mutant (IB43) exhibited significantly reduced survival across all stress conditions, with no evidence of cross-protection. Child et al. (2002) found that a Δ*rpoS* mutant of *S.* Typhimurium SL 1344 was more UV-C sensitive on Luria-Bertani agar than its wild-type counterpart. Similarly, Bucheli-Witschel et al. (2010) reported greater UV-C susceptibility in a Δ*rpoS* mutant of *E. coli* K12 in water. Our data further confirmed RpoS as a key regulator of adaptive stress responses and pathogen persistence in food systems.

Post-UV-C storage under temperature abuse revealed additional survival patterns. Heat-shocked *S. enterica* survived longer at elevated temperatures, likely due to the induction of RpoS-regulated chaperones and membrane-stabilizing proteins (Sirsat et al., 2015; Yoon et al., 2015). Desiccation-stressed cells exhibited brief population rebounds, possibly driven by the accumulation of osmoprotective solutes like trehalose and proline (Li et al., 2012; Chen and Meng, 2021). These findings illustrate that certain stresses may prime cells for recovery under fluctuating storage conditions. Importantly, temperature abuse—commonly encountered during cold chain breakdown—could unintentionally promote survival of sub-lethally stressed pathogens. In contrast, the Δ*rpoS* mutant showed rapid inactivation regardless of storage conditions, further underscoring the essential role of RpoS in cross-protection and persistence.

The stability of NRRL B-2354 across all tested conditions—including different food matrices, multiple sub-lethal stresses, and post-treatment storage scenarios such as temperature abuse—supports its use as a conservative and reliable surrogate for *S. enterica*. Its consistently equal or greater resistance to UV-C inactivation further reinforces its suitability, offering a safety margin critical for process validation. These findings align with previous reports highlighting its robustness in both thermal and non-thermal interventions (Jeong et al., 2011; Rane et al., 2021; Sudarsan and Keener, 2002), and extend its validation to novel conditions reflective of real-world food processing and storage environments.

A major strength—and key novelty—of this study lies in its comprehensive experimental design, which systematically integrated distinct food matrices, multiple sub-lethal stress conditions, and post-treatment storage scenarios to mimic real-world food processing and distribution environments. This multifactorial approach enables a more realistic evaluation of cross-protection mechanisms and microbial resistance during and after UV-C treatment. However, limitations include the reliance on culture-based methods, which may overlook viable but non-culturable (VBNC) cells, and the lack of molecular-level insight into stress response pathways. Future studies should apply transcriptomic, proteomic, or metabolomic tools to elucidate mechanisms of cross-protection and persistence, and evaluate combined interventions (e.g., UV-C combined with chemical sanitizers) across diverse commodities to strengthen food safety protocols. Moreover, although the inoculum levels used in this study were higher than typically found under natural contamination, they were selected to simulate a worst-case scenario, ensuring robust evaluation of UV-C efficacy against stressed populations and aligning with established practices in food safety challenge studies.

## 5 Conclusion

This study provides critical insights into how sub-lethal stress influences *S. enterica* survival during UV-C treatment on RWAs and FCLGs. Sub-lethal stresses enhanced UV-C resistance in *S. enterica* through cross-protection, an effect largely dependent on a functional *rpoS* gene. The Δ*rpoS* mutant (IB43) exhibited no cross-protection and was more susceptible to UV-C, confirming the key role of *rpoS* in stress adaptation. NRRL B-2354 showed comparable or greater resistance than *S. enterica*, supporting its use as a surrogate for UV-C validation. Tailing in survival curves suggests the presence of persister subpopulations, underscoring the need for hurdle-based sanitation strategies. Our findings highlight the importance of considering physiological heterogeneity in challenge studies. Incorporating sub-lethally stressed cells can improve predictive models and risk assessments. By integrating diverse stresses and food matrices, this study advances both mechanistic understanding and practical strategies for controlling foodborne pathogens.

## Data Availability

The original contributions presented in the study are included in the article/Supplementary material, further inquiries can be directed to the corresponding author.

## References

[ref1] Adhikari A. Syamaladevi R. M. Killinger K. Sablani S. S. (2015). Ultraviolet-C light inactivation of *Escherichia coli* O157:H7 and *Listeria monocytogenes* on organic fruit surfaces. Int. J. Food Microbiol. 210, 136–142. doi: 10.1016/j.ijfoodmicro.2015.06.018 26122954

[ref2] Almond Board of California (2014). Guidelines for using *Enterococcus faecium* NRRL B-2354 as a surrogate microorganism in almond process validation. Available online at: https://www.almonds.com/sites/default/files/guidelines_for_using_enterococcus_faecium_nrrl_b-2354_as_a_surrogate_microorganism_in_almond_process_validation.pdf (Accessed May 4, 2025).

[ref3] Bansal A. Jones T. M. Abd S. J. Danyluk M. D. Harris L. J. (2010). Most-probable-number determination of *Salmonella* levels in naturally contaminated raw almonds using two sample preparation methods. J. Food Protect. 73, 1986–1992. doi: 10.4315/0362-028X-73.11.1986 21219709

[ref5] Brandl M. T. Huynh S. (2014). Effect of the surfactant tween 80 on the detachment and dispersal of *Salmonella enterica* serovar Thompson single cells and aggregates from cilantro leaves as revealed by image analysis. Appl. Environ. Microbiol. 80, 5037–5042. doi: 10.1128/AEM.00795-14 24907336 PMC4135759

[ref6] Bucheli-Witschel M. Bassin C. Egli T. (2010). UV-C inactivation in *Escherichia coli* is affected by growth conditions preceding irradiation, in particular by the specific growth rate. J. Appl. Microbiol. 109, 1733–1744. doi: 10.1111/j.1365-2672.2010.04802.x, PMID: 20629801

[ref7] Calle A. Fernandez M. Montoya B. Schmidt M. Thompson J. (2021). UV-C LED irradiation reduces *Salmonella* on chicken and food contact surfaces. Food Secur. 10:1459. doi: 10.3390/foods10071459, PMID: 34202557 PMC8305569

[ref8] Capozzi V. Fiocco D. Amodio M. L. Gallone A. Spano G. (2009). Bacterial stressors in minimally processed food. Int. J. Mol. Sci. 10, 3076–3105. doi: 10.3390/ijms10073076, PMID: 19742126 PMC2738913

[ref9] Carey C. M. Kostrzynska M. Thompson S. (2009). *Escherichia coli* O157:H7 stress and virulence gene expression on Romaine lettuce using comparative real-time PCR. J. Microbiol. Methods 77, 235–242. doi: 10.1016/j.mimet.2009.02.010, PMID: 19248811

[ref10] CDC (2004). Outbreak of Salmonella serotype Enteritidis infections associated with raw almonds-United States and Canada, 2003-2004. Available online at: https://www.cdc.gov/mmwr/preview/mmwrhtml/mm5322a8.htm (Accessed May 4, 2025). 15190247

[ref11] CDC (2024). Reports of selected Salmonella outbreak investigations. Available online at: https://www.cdc.gov/salmonella/outbreaks.html (Accessed May 4, 2025).

[ref12] Cerf O. Métro F. (1977). Tailing of survival curves of *Bacillus licheniformis* spores treated with hydrogen peroxide. J. Appl. Bacteriol. 42, 405–415. doi: 10.1111/j.1365-2672.1977.tb00708.x, PMID: 885821

[ref13] Chaves R. D. Aspridou Z. Sant'Ana A. S. Koutsoumanis K. P. (2019). Effect of chlorine stress on the subsequent growth behavior of individual *Salmonella* cells. Food Res. Int. 123, 311–316. doi: 10.1016/j.foodres.2019.05.006, PMID: 31284981

[ref15] Chen Z. Meng J. (2021). Persistence of *Salmonella enterica* and *Enterococcus faecium* NRRL B-2354 on baby spinach subjected to temperature abuse after exposure to sub-lethal stresses. Food Secur. 10:2141. doi: 10.3390/foods10092141, PMID: 34574255 PMC8472226

[ref16] Child M. Strike P. Pickup R. Edwards C. (2002). *Salmonella* Typhimurium displays cyclical patterns of sensitivity to UV-C killing during prolonged incubation in the stationary phase of growth. FEMS Microbiol. Lett. 213, 81–85. doi: 10.1111/j.1574-6968.2002.tb11289.x, PMID: 12127492

[ref17] Coroller L. Leguérinel I. Mettler E. Savy N. Mafart P. (2006). General model, based on two mixed Weibull distributions of bacterial resistance, for describing various shapes of inactivation curves. Appl. Environ. Microbiol. 72, 6493–6502. doi: 10.1128/AEM.00876-06 17021197 PMC1610288

[ref18] Derossi A. Fiore A. G. De Pilli T. Severini C. (2011). A review on acidifying treatments for vegetable canned food. Crit. Rev. Food Sci. Nutr. 51, 955–964. doi: 10.1080/10408398.2010.491163, PMID: 21955094

[ref19] Dexter E. Rollwagen-Bollens G. Bollens S. M. (2018). The trouble with stress: a flexible method for the evaluation of nonmetric multidimensional scaling. Limnol. Oceanogr. Methods 16, 434–443. doi: 10.1002/lom3.10257

[ref20] Dhakal J. Sharma C. S. Nannapaneni R. McDANIEL C. D. Kim T. Kiess A. (2019). Effect of chlorine-induced sub-lethal oxidative stress on the biofilm-forming ability of *Salmonella* at different temperatures, nutrient conditions, and substrates. J. Food Protect. 82, 78–92. doi: 10.4315/0362-028X.JFP-18-119 30586327

[ref21] Escalona V. H. Aguayo E. Martínez-Hernández G. B. Artés F. (2010). UV-C doses to reduce pathogen and spoilage bacterial growth *in vitro* and in baby spinach. Postharvest Biol. Technol. 56, 223–231. doi: 10.1016/j.postharvbio.2010.01.008

[ref22] Estilo E. E. C. Gabriel A. A. (2017). Previous stress exposures influence subsequent UV-C resistance of *Salmonella enterica* in coconut liquid endosperm. LWT 86, 139–147. doi: 10.1016/j.lwt.2017.07.061

[ref23] Fan X. Huang R. Chen H. (2017). Application of ultraviolet C technology for surface decontamination of fresh produce. Trends Food Sci. Technol. 70, 9–19. doi: 10.1016/j.tifs.2017.10.004

[ref24] FDA (2000). Part 179 irradiation in the production, processing and handling of food. Sec. 179.39 ultraviolet radiation for the processing and treatment of food. Available online at: https://www.ecfr.gov/current/title-21/chapter-I/subchapter-B/part-179 (Accessed May 4, 2025).

[ref25] FDA (2014). Sanitary transportation of human and animal food. Available online at: https://www.federalregister.gov/documents/2016/04/06/2016-07330/sanitary-transportation-of-human-and-animal-food (Accessed May 4, 2025).

[ref26] FDA. (2017). Food code 2017. Available online at: https://www.fda.gov/food/fda-food-code/food-code-2017 (Accessed May 4, 2025).

[ref27] Foster J. W. Spector M. P. (1995). How *Salmonella* survive against the odds. Ann. Rev. Microbiol. 49, 145–174. doi: 10.1146/annurev.mi.49.100195.001045 8561457

[ref28] Fukuzaki S. (2006). Mechanisms of actions of sodium hypochlorite in cleaning and disinfection processes. Biocontrol Sci. 11, 147–157. doi: 10.4265/bio.11.147, PMID: 17190269

[ref29] Gabriel A. A. (2015). Previous physicochemical stress exposures influence subsequent resistance of *Escherichia coli* O157:H7, *Salmonella enterica*, and *Listeria monocytogenes* to ultraviolet-C in coconut liquid endosperm beverage. Int. J. Food Microbiol. 201, 7–16. doi: 10.1016/j.ijfoodmicro.2015.02.003, PMID: 25723813

[ref30] Gao M. Tang J. Villa-Rojas R. Wang Y. Wang S. (2011). Pasteurization process development for controlling *Salmonella* in in-shell almonds using radio frequency energy. J. Food Eng. 104, 299–306. doi: 10.1016/j.jfoodeng.2010.12.021

[ref31] Ge C. Bohrerova Z. Lee J. (2013). Inactivation of internalized *Salmonella* Typhimurium in lettuce and green onion using ultraviolet C irradiation and chemical sanitizers. J. Appl. Microbiol. 114, 1415–1424. doi: 10.1111/jam.12154, PMID: 23351161

[ref32] Geeraerd A. H. Herremans C. H. Van Impe J. F. (2000). Structural model requirements to describe microbial inactivation during a mild heat treatment. Int. J. Food Microbiol. 59, 185–209. doi: 10.1016/S0168-1605(00)00362-7, PMID: 11020040

[ref33] Geeraerd A. H. Valdramidis V. P. Van Impe J. F. (2005). GInaFiT, a freeware tool to assess non-log-linear microbial survivor curves. Int. J. Food Microbiol. 102, 95–105. doi: 10.1016/j.ijfoodmicro.2004.11.038, PMID: 15893399

[ref34] Goodburn C. Wallace C. A. (2013). The microbiological efficacy of decontamination methodologies for fresh produce: a review. Food Control 32, 418–427. doi: 10.1016/j.foodcont.2012.12.012

[ref35] Gunter-Ward D. M. Patras A. Bhullar M. S. Kilonzo-Nthenge A. Pokharel B. Sasges M. (2018). Efficacy of ultraviolet (UV-C) light in reducing foodborne pathogens and model viruses in skim milk. J. Food Process. Preserv. 42:e13485. doi: 10.1111/jfpp.13485

[ref36] Herman K. M. Hall A. J. Gould L. H. (2015). Outbreaks attributed to fresh leafy vegetables, United States, 1973-2012. Epidemiol. Infect. 143, 3011–3021. doi: 10.1017/S0950268815000047, PMID: 25697407 PMC4591532

[ref37] Hu M. Gurtler J. B. (2017). Selection of surrogate bacteria for use in food safety challenge studies: a review. J. Food Prot. 80, 1506–1536. doi: 10.4315/0362-028X.JFP-16-536 28805457

[ref38] Huang J. Luo Y. Zhou B. Zheng J. Nou X. (2019). Growth and survival of *Salmonella enterica* and *Listeria monocytogenes* on fresh-cut produce and their juice extracts: impacts and interactions of food matrices and temperature abuse conditions. Food Control 100, 300–304. doi: 10.1016/j.foodcont.2018.12.035

[ref39] Isaacs S. Aramini J. Ciebin B. Farrar J. A. Ahmed R. Middleton D.. (2005). An international outbreak of salmonellosis associated with raw almonds contaminated with a rare phage type of *Salmonella* Enteritidis. J. Food Prot. 68, 191–198. doi: 10.4315/0362-028X-68.1.191 15690826

[ref40] Jeong S. Marks B. P. Ryser E. T. (2011). Quantifying the performance of *Pediococcus* sp. (NRRL B-2354: *Enterococcus faecium*) as a nonpathogenic surrogate for *Salmonella* Enteritidis PT30 during moist-air convection heating of almonds. J. Food Prot. 74, 603–609. doi: 10.4315/0362-028X.JFP-10-416 21477474

[ref41] Jimenez L. R. Hall W. A. IV Rodriquez M. S. Cooper W. J. Muhareb J. Jones T.. (2015). Quantifying residues from postharvest propylene oxide fumigation of almonds and walnuts. J. AOAC Int. 98, 1423–1427. doi: 10.5740/jaoacint.14-199, PMID: 26525262

[ref42] Kopit L. M. Kim E. B. Siezen R. J. Harris L. J. Marco M. L. (2014). Safety of the surrogate microorganism *Enterococcus faecium* NRRL B-2354 for use in thermal process validation. Appl. Environ. Microbiol. 80, 1899–1909. doi: 10.1128/AEM.03859-13 24413604 PMC3957640

[ref44] Koutsoumanis K. P. Sofos J. N. (2004). Comparative acid stress response of *Listeria monocytogenes*, *Escherichia coli* O157:H7 and *Salmonella* Typhimurium after habituation at different pH conditions. Lett. Appl. Microbiol. 38, 321–326. doi: 10.1111/j.1472-765X.2004.01491.x, PMID: 15214733

[ref46] Li H. Bhaskara A. Megalis C. Tortorello M. L. (2012). Transcriptomic analysis of *Salmonella* desiccation resistance. Foodborne Pathog. Dis. 9, 1143–1151. doi: 10.1089/fpd.2012.1254 23237410

[ref47] López-Gálvez F. Allende A. Truchado P. Martínez-Sánchez A. Tudela J. A. Selma M. V.. (2010). Suitability of aqueous chlorine dioxide versus sodium hypochlorite as an effective sanitizer for preserving quality of fresh-cut lettuce while avoiding by-product formation. Postharvest Biol. Technol. 55, 53–60. doi: 10.1016/j.postharvbio.2009.08.001

[ref48] Mafart P. Couvert O. Gaillard S. Leguérinel I. (2002). On calculating sterility in thermal preservation methods: application of the Weibull frequency distribution model. Int. J. Food Microbiol. 72, 107–113. doi: 10.1016/S0168-1605(01)00624-9, PMID: 11843401

[ref49] Mukhopadhyay S. Ukuku D. O. Juneja V. Fan X. J. F. C. (2014). Effects of UV-C treatment on inactivation of *Salmonella enterica* and *Escherichia coli* O157:H7 on grape tomato surface and stem scars, microbial loads, and quality. Food Control 44, 110–117. doi: 10.1016/j.foodcont.2014.03.027

[ref50] Mutz Y. S. Rosario D. K. Bernardes P. C. Paschoalin V. M. Conte-Junior C. A. (2020). Modeling *Salmonella* Typhimurium inactivation in dry-fermented sausages: previous habituation in the food matrix undermines UV-C decontamination efficacy. Front. Microbiol. 11:591. doi: 10.3389/fmicb.2020.00591 32322246 PMC7156554

[ref51] National Advisory Committee on Microbiological Criteria for Foods (2010). Parameters for determining inoculated pack/challenge study protocols. J. Food Prot. 73, 140–202. doi: 10.4315/0362-028X-73.1.140 20051217

[ref52] Ndraha N. Hsiao H. I. Vlajic J. Yang M. F. Lin H. T. V. (2018). Time-temperature abuse in the food cold chain: review of issues, challenges, and recommendations. Food Control 89, 12–21. doi: 10.1016/j.foodcont.2018.01.027

[ref53] Oksanen J. (2013). Vegan: ecological diversity. R project. 368, 1–11. Available online at: https://mirror.linux.duke.edu/cran/web/packages/vegan/vignettes/diversity-vegan.pdf (Accessed May 4, 2025).

[ref55] Rane B. Lacombe A. Sablani S. Bridges D. F. Tang J. Guan J.. (2021). Effects of moisture content and mild heat on the ability of gaseous chlorine dioxide against *Salmonella* and *Enterococcus faecium* NRRL B-2354 on almonds. Food Control 123:107732. doi: 10.1016/j.foodcont.2020.107732

[ref56] Ruiz-Hernández K. Ramírez-Rojas N. Z. Meza-Plaza E. F. García-Mosqueda C. Jauregui-Vázquez D. Rojas-Laguna R.. (2021). UV-C treatments against *Salmonella* Typhimurium ATCC 14028 in inoculated peanuts and almonds. Food Eng. Rev. 13, 706–712. doi: 10.1007/s12393-020-09272-7

[ref57] Samelis J. Sofos J. N. (2002). “Strategies to control stress-adapted pathogens” in Microbial stress adaptation and food safety. eds. Yousef A. E. Juneja V. K. (Boca Raton, FL: CRC Press), 303–351.

[ref58] Singh R. Jiang X. Luo F. (2010). Thermal inactivation of heat-shocked *Escherichia coli* O157:H7, *Salmonella*, and *Listeria monocytogenes* in dairy compost. J. Food Protect. 73, 1633–1640. doi: 10.4315/0362-028X-73.9.1633 20828469

[ref59] Sirsat S. A. Baker C. A. Park S. H. Muthaiyan A. Dowd S. E. Ricke S. C. (2015). Transcriptomic response of *Salmonella* Typhimurium heat shock gene expression under thermal stress at 48 C. J. Food Res. 4:51. doi: 10.5539/jfr.v4n5p51

[ref60] Smith J. L. Benedict R. C. Palumbo S. A. (1982). Protection against heat-injury in *Staphylococcus aureus* by solutes. J. Food Prot. 45, 54–58. 30866350 10.4315/0362-028X-45.1.54

[ref61] Sudarsan A. Keener K. M. (2002). Inactivation of *Salmonella enterica* serovars and *Escherichia coli* O157:H7 surrogate from baby spinach leaves using high voltage atmospheric cold plasma (HVACP). LWT 155:112903. doi: 10.1016/j.lwt.2021.112903

[ref62] Wesche A. M. Gurtler J. B. Marks B. P. Ryser E. T. (2009). Stress, sublethal injury, resuscitation, and virulence of bacterial foodborne pathogens. J. Food Prot. 72, 1121–1138. doi: 10.4315/0362-028X-72.5.1121 19517746

[ref63] Wickham H. (2011). ggplot2 Wiley Interdiscip. Rev. Comput. Stat. 3, 180–185. doi: 10.1002/wics.147

[ref64] Yoon Y. Lee H. Lee S. Kim S. Choi K. H. (2015). Membrane fluidity-related adaptive response mechanisms of foodborne bacterial pathogens under environmental stresses. Food Res. Int. 72, 25–36. doi: 10.1016/j.foodres.2015.03.016

[ref65] Yun J. Yan R. Fan X. Gurtler J. Phillips J. (2013). Fate of *E. coli* O157:H7, *Salmonella* spp. and potential surrogate bacteria on apricot fruit, following exposure to UV-C light. Int. J. Food Microbiol. 166, 356–363. doi: 10.1016/j.ijfoodmicro.2013.07.021, PMID: 24021820

