# Supplementary material for: Sub-lethal stress-induced cross-protection against ultraviolet-C in Salmonella enterica on raw whole almonds and fresh-cut leafy greens
Source: Front Microbiol. 2025 Jun 18;16:1599380. doi: 10.3389/fmicb.2025.1599380 (PMC12213592; doi:10.3389/fmicb.2025.1599380)
Supplement: Supplementary file 1 [file Data_Sheet_1.docx]

Supplementary Material

# Supplementary Results and Discussion

# Mathematical modeling

## RWAs

The six models we evaluated adequately represented the survival curves, effectively capturing the prolonged tail phase for both unstressed and stressed cells (Tables S1–S6). Each model showed robust performance, with high average adjusted *R*^2^ values (≥0.93) and low RMSE values (≤0.20), except log-linear with tail, which had an average adjusted *R*^2^ of 0.86 and RMSE of 0.29. Among the models, double Weibull provided the best fit for overall survival curves, with an average adjusted *R*^2^ of 0.96 and RMSE of 0.12, followed closely by log-linear with shoulder and tail (adjusted *R*^2^: 0.96, RMSE: 0.14) and biphasic (adjusted *R*^2^: 0.95, RMSE: 0.19). Weibull and biphasic with shoulder also performed well, with adjusted *R*^2^ values of 0.93 and RMSE values of 0.18 and 0.20, respectively.

Weibull assumes variable resistance among cells in a population, treating survival as a cumulative effect of lethal events (Peleg and Cole, 1998). Acid-stressed *S. enterica* required the longest time for the first decimal reduction (*δ* = 51.22), while oxidation-stressed cells showed the quickest reduction (*δ* = 0.09) (Table S1). NRRL B-2354 required longer times for the first decimal reduction than *S. enterica* under stress. The shape factor (*p*) in Weibull has no specific biological interpretation, though it can still relate to survival curve shapes. In this study, all survival curves showed upward concavity (*p* < 1), indicating tailing, independent of microorganism or stress type.

Double Weibull, a modification of Weibull, assumes two subpopulations with distinct resistance levels (Coroller et al., 2006). This model effectively captured changes in curve shape across conditions (Fig. 1–3 and Table S2). For instance, oxidation-stressed *S. enterica*, IB43, and NRRL B-2354 showed marked differences between sensitive and resistant subpopulations, with shorter times to the first decimal reduction for the sensitive subpopulation. NRRL B-2354 had comparable or higher *α* values than *S. enterica*, highlighting its potential as a suitable surrogate.

Log-linear with tail, as well as log-linear with shoulder and tail, captured log-linear inactivation phases and smooth transitions between phases (Geeraerd et al., 2005). For all microorganisms, the lowest log(*N*_res_) values were noticed in oxidation-stressed cells, while acid-stressed cells had relatively high values (Tables S3 and S4). Notably, heat-shocked NRRL B-2354 had a prolonged shoulder (SI: 7.50 min), reflecting more viable cells at the end of UV-C exposure. The rate constant *k*_max_ showed no dependence on microorganism or stress type.

Biphasic, similar to double Weibull, presumes two distinct subpopulations with different resistance levels (Cerf and Métro, 1977). In this study, survival curves consistently showed two inactivation rates, highlighting the transition of cells from a more sensitive to a more resistant state (Table S5). The prominence of tailing was reflected in the lower *k*_max2_ (resistant subpopulation) compared to *k*_max1_ (sensitive subpopulation), with oxidation-stressed cells having the highest *k*_max1_ and *k*_max2_ values among all stress conditions. NRRL B-2354 showed similar or lower *k*_max1_ and *k*_max2_ values than *S. enterica* across all stresses except acid stress. Tailing was also observed through the parameter *f* in biphasic modeling, where higher *f* values indicated a larger sensitive subpopulation, especially in oxidation-stressed or heat-shocked cells. NRRL B-2354 had similar or lower *f* values than *S. enterica* after stress, with the exception of unstressed cells, where NRRL B-2354 had higher *f* values.

Biphasic with shoulder, introduced by Geeraerd et al. (2005), accounts for an initial shoulder followed by a biphasic pattern. Similar *f*, *k*_max1_, and *k*_max2_ values were obtained when applying both biphasic and biphasic with shoulder (Tables S5 and S6). A shoulder phase was present only in heat-shocked, oxidation-stressed, or acid-stressed NRRL B-2354 (SI values: 0.93, 1.65, and 7.81 min, respectively), suggesting initial UV-C resistance. This makes NRRL B-2354 an appropriate surrogate for validating UV-C treatments aimed at inactivating *S. enterica* on RWAs.

## FCLGs

The six models effectively captured the inactivation dynamics of *S. enterica*, IB43, and NRRL B-2354 on FCLGs under UV-C treatment, including tailing and shoulder phases for both unstressed and stressed cells (Tables S1–S6). The high adjusted *R*^2^ values (≥0.85) and low RMSE values (≤0.55) of each model support their reliability.

Weibull provided a strong fit with an average adjusted *R*^2^ of 0.87 and RMSE of 0.55 (Table S1). This model captured the upward concavity, indicating tailing effects (*p* < 1) across all survival curves. Among the stress conditions, oxidation-stressed *S. enterica* exhibited the highest resistance, taking the longest time for the first decimal reduction (*δ* = 4.63), while heat-shocked or acid-stressed cells were inactivated fastest (*δ* = 0.03). IB43, lacking functional *rpoS*, showed lower *δ* values across all conditions, reflecting higher UV-C sensitivity. For NRRL B-2354, desiccation-stressed, heat-shocked, or acid-stressed cells displayed higher *δ* values than *S. enterica* and IB43, underscoring the robustness of NRRL B-2354 as a surrogate.

Double Weibull offered the best overall fit, with an average adjusted *R*^2^ of 0.95 and RMSE of 0.32 (Table S2). This model successfully captured the dual subpopulations of sensitive and resistant cells. Oxidation-stressed *S. enterica* showed similar *δ*_1_ and *δ*_2_ values, indicating uniform resistance, while heat-shocked or acid-stressed cells exhibited high *δ*_2_ values. The absence of *rpoS* in IB43 resulted in consistently lower *δ*_2_ values, confirming greater sensitivity. NRRL B-2354 had lower *α* values than *S. enterica* under most stresses, except for oxidation, suggesting a more gradual shift between sensitive and resistant subpopulations.

Log-linear with tail performed moderately well, with an average adjusted *R*^2^ of 0.85 and RMSE of 0.55 (Table S3). It was particularly effective in capturing tailing for stressed cells, with oxidation-stressed *S. enterica* exhibiting the highest log(*N*_res_) values, indicating greater survival in the tail phase. For IB43, quicker inactivation (higher *k*_max_) and shorter tails (lower log(*N*_res_)) reflected its UV-C sensitivity. NRRL B-2354 had similar or higher log(*N*_res_) values compared to *S. enterica*, indicating comparable or better persistence in the tail phase.

Log-linear with shoulder and tail provided a solid fit, with an average adjusted *R*^2^ of 0.94 and RMSE of 0.33 (Table S4). This model captured differences in initial resistance and inactivation rates across stress conditions. Heat-shocked *S. enterica* displayed the highest residual populations [log(*N*_res_)] but showed faster inactivation rates (*k*_max_). IB43 had shorter shoulder phases across all conditions, highlighting its increased UV-C sensitivity due to the lack of functional *rpoS*. NRRL B-2354 demonstrated comparable or higher residuals than *S. enterica*.

Biphasic effectively described inactivation patterns, capturing distinct behaviors of sensitive and resistant subpopulations, with an average adjusted *R*^2^ of 0.90 and RMSE of 0.50 (Table S5). Acid-stressed *S. enterica* had higher initial inactivation rates (*k*_max1_), suggesting rapid reduction of the more susceptible cells. IB43 showed faster inactivation under most conditions, with higher *k*_max1_ values than *S. enterica* (except for acid stress), consistent with its increased sensitivity from the absence of *rpoS*. The similar or slower initial inactivation rates of NRRL B-2354 indicated comparable or greater resistance in its sensitive subpopulation, reinforcing its suitability as a surrogate.

Finally, biphasic with shoulder provided reliable descriptions of survival data, with an average adjusted *R*^2^ of 0.92 and RMSE of 0.37 (Table S6). This model captured the presence of an initial shoulder phase, reflecting variability in resistance across cells. Higher *k*_max1_ values in acid-stressed *S. enterica* reflected rapid inactivation of the sensitive subpopulation. Across all conditions, IB43 displayed larger proportions of the sensitive subpopulation (higher *f* values) due to its reduced resistance from *rpoS* deletion. NRRL B-2354, in contrast, had similar or extended shoulder phases, which indicates comparable or delayed inactivation onset, affirming its robustness as a surrogate.

## Comparison between RWAs and FCLGs

The lower *δ* values in Weibull for FCLGs compared to RWAs indicated quicker inactivation on FCLGs (Table S1). Double Weibull highlights a more pronounced difference between *δ*_1_ and *δ*_2_ on RWAs than on FCLGs (Table S2), suggesting greater variability in resistance levels on RWAs. In log-linear with tail (Table S3), higher *k*_max_ values for FCLGs indicated faster inactivation rates on FCLGs compared to RWAs, while RWAs displayed a more prominent tailing effect, as reflected in higher log(*N*_res_) values. Log-linear with shoulder and tail showed that survival curves for FCLGs had higher inactivation rates, shorter shoulder phases, and lower residual populations than those for RWAs (Table S4). Both biphasic models revealed higher *k*_max1_ and *k*_max2_ values for FCLGs (Tables S5 and S6), indicating quicker inactivation of both sensitive and resistant subpopulations. In contrast, RWAs, with larger proportions of resistant cells, may provide a more protective niche for bacterial survival.

**References**

Cerf, O., & Métro, F. (1977). Tailing of survival curves of *Bacillus licheniformis* spores treated with hydrogen peroxide. J. Appl. Bacteriol. 42, 405-415. doi: 10.1111/j.1365-2672.1977.tb00708.x

Coroller, L., Leguérinel, I., Mettler, E., Savy, N., & Mafart, P. (2006). General model, based on two mixed Weibull distributions of bacterial resistance, for describing various shapes of inactivation curves. Appl. Environ. Microbiol. 72, 6493-6502. doi: 10.1128/AEM.00876-06

Geeraerd, A. H., Valdramidis, V. P., & Van Impe, J. F. (2005). GInaFiT, a freeware tool to assess non-log-linear microbial survivor curves. Int. J. Food Microbiol. 102, 95-105. doi: 10.1016/j.ijfoodmicro.2004.11.038

Peleg, M., & Cole, M. B. (1998). Reinterpretation of microbial survival curves. Crit. Rev. Food Sci. Nutr. 38, 353-380. doi: 10.1080/10408699891274246

# Supplementary Tables

**Table S1** Parameter estimates and goodness-of-fit of Weibull for the survival of *Salmonella enterica*, *S.* Typhimurium IB43, and *Enterococcus faecium* NRRL B-2354 on raw whole almonds (RWAs) and fresh-cut leafy greens (FCLGs) during ultraviolet-C (UV-C) treatment, with or without prior exposure to sub-lethal stress.

| Food matrix | Stress | Microorganism | Parameter estimate^a^ | | Goodness-of-fit | |
| --- | --- | --- | --- | --- | --- | --- |
|  |  |  | *δ* | *p* | Adjusted *R*^2^ | RMSE^b^ |
| RWAs | No stress | *S. enterica* | 15.15 | 0.50 | 0.93 | 0.18 |
|  |  | IB43^c^ | 4.21 | 0.32 | 0.96 | 0.16 |
|  |  | NRRL B-2354 | 11.28 | 0.44 | 0.97 | 0.12 |
|  | Desiccation | *S. enterica* | 1.52 | 0.08 | 0.99 | 0.04 |
|  |  | IB43 | 2.26 | 0.34 | 0.98 | 0.13 |
|  |  | NRRL B-2354 | 120.55 | 0.32 | 0.88 | 0.09 |
|  | Heat shock | *S. enterica* | 0.27 | 0.17 | 0.98 | 0.12 |
|  |  | IB43 | 0.30 | 0.19 | 0.95 | 0.19 |
|  |  | NRRL B-2354 | 9.79 | 0.53 | 0.80 | 0.42 |
|  | Oxidation | *S. enterica* | 0.09 | 0.19 | 0.96 | 0.20 |
|  |  | IB43 | 0.01 | 0.18 | 0.98 | 0.20 |
|  |  | NRRL B-2354 | 6.63 | 0.55 | 0.88 | 0.42 |
|  | Acid | *S. enterica* | 51.22 | 0.31 | 0.90 | 0.11 |
|  |  | IB43 | 2.38 | 0.26 | 0.89 | 0.24 |
|  |  | NRRL B-2354 | 101.87 | 0.30 | 0.89 | 0.09 |
|  | Average |  |  |  | 0.93 | 0.18 |
| FCLGs | No stress | *S. enterica* | 2.61 | 0.51 | 0.92 | 0.51 |
|  |  | IB43 | 0.11 | 0.39 | 0.96 | 0.47 |
|  |  | NRRL B-2354 | 1.20 | 0.28 | 0.75 | 0.50 |
|  | Desiccation | *S. enterica* | 0.53 | 0.22 | 0.74 | 0.72 |
|  |  | IB43 | 0.16 | 0.42 | 0.95 | 0.49 |
|  |  | NRRL B-2354 | 0.84 | 0.33 | 0.82 | 0.66 |
|  | Heat shock | *S. enterica* | 0.03 | 0.18 | 0.84 | 0.53 |
|  |  | IB43 | 0.07 | 0.34 | 0.87 | 0.85 |
|  |  | NRRL B-2354 | 0.05 | 0.18 | 0.81 | 0.58 |
|  | Oxidation | *S. enterica* | 4.63 | 0.42 | 0.95 | 0.21 |
|  |  | IB43 | 0.15 | 0.41 | 0.94 | 0.54 |
|  |  | NRRL B-2354 | 1.91 | 0.37 | 0.94 | 0.29 |
|  | Acid | *S. enterica* | 0.03 | 0.17 | 0.90 | 0.40 |
|  |  | IB43 | 0.09 | 0.36 | 0.83 | 1.03 |
|  |  | NRRL B-2354 | 0.31 | 0.20 | 0.90 | 0.36 |
|  | Average |  |  |  | 0.87 | 0.55 |

^a^*δ*, time of the first decimal reduction (min); *p*, shape factor.

^b^RMSE, root mean square error.

^c^IB43 is the Δ*rpoS* mutant of *S.* Typhimurium ATCC 14028.

**Table S2** Parameter estimates and goodness-of-fit of double Weibull for the survival of *Salmonella enterica*, *S.* Typhimurium IB43, and *Enterococcus faecium* NRRL B-2354 on raw whole almonds (RWAs) and fresh-cut leafy greens (FCLGs) during ultraviolet-C (UV-C) treatment, with or without prior exposure to sub-lethal stress.

| Food matrix | Stress | Microorganism | Parameter estimate^a^ | | | | Goodness-of-fit | |
| --- | --- | --- | --- | --- | --- | --- | --- | --- |
|  |  |  | *α* | *δ*_1_ | *δ*_2_ | *p* | Adjusted *R*^2^ | RMSE^b^ |
| RWAs | No stress | *S. enterica* | 2.00 | 14.69 | 193.52 | 0.52 | 0.91 | 0.20 |
|  |  | IB43^c^ | 1.11 | 3.20 | 51.86 | 0.74 | 0.98 | 0.11 |
|  |  | NRRL B-2354 | 2.10 | 11.28 | 11.28 | 0.44 | 0.96 | 0.13 |
|  | Desiccation | *S. enterica* | 1.40 | 0.83 | 0.98 | 0.08 | 0.99 | 0.04 |
|  |  | IB43 | 2.47 | 2.86 | 263.28 | 0.43 | 0.99 | 0.09 |
|  |  | NRRL B-2354 | 0.00 | 120.55 | 120.55 | 0.32 | 0.85 | 0.10 |
|  | Heat shock | *S. enterica* | 2.10 | 0.27 | 0.27 | 0.17 | 0.97 | 0.14 |
|  |  | IB43 | 1.51 | 0.68 | 47.78 | 0.41 | 0.95 | 0.18 |
|  |  | NRRL B-2354 | 1.87 | 11.40 | 101.82 | 2.58 | 1.00 | 0.05 |
|  | Oxidation | *S. enterica* | 3.29 | 0.07 | 128689.53 | 0.20 | 0.97 | 0.17 |
|  |  | IB43 | 4.30 | 0.03 | 15542.07 | 0.20 | 0.98 | 0.21 |
|  |  | NRRL B-2354 | 3.60 | 2.05 | 156673.52 | 0.45 | 0.98 | 0.15 |
|  | Acid | *S. enterica* | 0.84 | 12.07 | 661263986579489.00 | 0.77 | 0.98 | 0.04 |
|  |  | IB43 | 1.36 | 2.86 | 82.27 | 0.85 | 0.98 | 0.21 |
|  |  | NRRL B-2354 | 0.00 | 101.87 | 101.87 | 0.30 | 0.86 | 0.10 |
|  | Average |  |  |  |  |  | 0.96 | 0.12 |
| FCLGs | No stress | *S. enterica* | 4.80 | 1.97 | 28208.60 | 0.57 | 0.99 | 0.21 |
|  |  | IB43 | 5.90 | 0.14 | 138.10 | 0.42 | 0.92 | 0.65 |
|  |  | NRRL B-2354 | 1.92 | 3.48 | 67.95 | 3.63 | 0.99 | 0.11 |
|  | Desiccation | *S. enterica* | 3.97 | 0.05 | 0.66 | 0.21 | 0.90 | 0.44 |
|  |  | IB43 | 1.72 | 0.07 | 0.07 | 0.36 | 0.93 | 0.60 |
|  |  | NRRL B-2354 | 3.92 | 1.57 | 1104.71 | 0.62 | 0.97 | 0.27 |
|  | Heat shock | *S. enterica* | 3.69 | 0.21 | 100000000000000000000.00 | 0.41 | 0.98 | 0.16 |
|  |  | IB43 | 5.70 | 0.06 | 6291.20 | 0.40 | 0.99 | 0.21 |
|  |  | NRRL B-2354 | 3.61 | 0.30 | 5962.76 | 0.47 | 0.97 | 0.24 |
|  | Oxidation | *S. enterica* | 3.20 | 4.63 | 4.63 | 0.42 | 0.94 | 0.24 |
|  |  | IB43 | 5.70 | 0.15 | 0.15 | 0.41 | 0.88 | 0.76 |
|  |  | NRRL B-2354 | 3.80 | 1.91 | 1.91 | 0.37 | 0.92 | 0.33 |
|  | Acid | *S. enterica* | 3.70 | 0.04 | 14981418.09 | 0.24 | 0.99 | 0.14 |
|  |  | IB43 | 5.60 | 0.36 | 2139.95 | 0.72 | 0.99 | 0.03 |
|  |  | NRRL B-2354 | 2.00 | 0.03 | 0.03 | 0.17 | 0.93 | 0.28 |
|  | Average |  |  |  |  |  | 0.95 | 0.32 |

^a^*α*, difference between the sensitive subpopulation and the resistant subpopulation (log CFU/sample unit); *δ*_1_, time of the first decimal reduction of the sensitive subpopulation (min); *δ*_2_, time of the first decimal reduction of the resistant subpopulation (min); *p*, shape factor.

^b^RMSE, root mean square error.

^c^IB43 is the Δ*rpoS* mutant of *S.* Typhimurium ATCC 14028.

**Table S3** Parameter estimates and goodness-of-fit of log-linear with tail for the survival of *Salmonella enterica*, *S.* Typhimurium IB43, and *Enterococcus faecium* NRRL B-2354 on raw whole almonds (RWAs) and fresh-cut leafy greens (FCLGs) during ultraviolet-C (UV-C) treatment, with or without prior exposure to sub-lethal stress.

| Food matrix | Stress | Microorganism | Parameter estimate^a^ | | Goodness-of-fit | |
| --- | --- | --- | --- | --- | --- | --- |
|  |  |  | *k*_max_ | log(*N*_res_) | Adjusted *R*^2^ | RMSE^b^ |
| RWAs | No stress | *S. enterica* | 0.12 | 3.64 | 0.89 | 0.22 |
|  |  | IB43^c^ | 0.07 | 5.12 | 0.68 | 0.43 |
|  |  | NRRL B-2354 | 0.14 | 3.52 | 0.93 | 0.18 |
|  | Desiccation | *S. enterica* | 3.11 | 4.37 | 0.95 | 0.10 |
|  |  | IB43 | 0.28 | 3.21 | 0.90 | 0.31 |
|  |  | NRRL B-2354 | 0.20 | 4.94 | 0.98 | 0.03 |
|  | Heat shock | *S. enterica* | 0.95 | 3.67 | 0.71 | 0.42 |
|  |  | IB43 | 1.02 | 3.36 | 0.83 | 0.34 |
|  |  | NRRL B-2354 | 0.25 | 3.66 | 0.94 | 0.24 |
|  | Oxidation | *S. enterica* | 0.92 | 2.57 | 0.74 | 0.55 |
|  |  | IB43 | 1.20 | 1.83 | 0.72 | 0.73 |
|  |  | NRRL B-2354 | 0.32 | 2.45 | 0.92 | 0.34 |
|  | Acid | *S. enterica* | 0.19 | 4.70 | 0.98 | 0.05 |
|  |  | IB43 | 0.61 | 3.95 | 0.86 | 0.28 |
|  |  | NRRL B-2354 | 0.29 | 4.92 | 0.93 | 0.07 |
|  | Average |  |  |  | 0.86 | 0.29 |
| FCLGs | No stress | *S. enterica* | 0.41 | 1.04 | 0.95 | 0.38 |
|  |  | IB43 | 2.01 | 0.02 | 0.85 | 0.88 |
|  |  | NRRL B-2354 | 0.95 | 3.35 | 0.89 | 0.34 |
|  | Desiccation | *S. enterica* | 1.33 | 2.01 | 0.92 | 0.39 |
|  |  | IB43 | 1.95 | 0.04 | 0.80 | 1.00 |
|  |  | NRRL B-2354 | 0.66 | 1.73 | 0.95 | 0.36 |
|  | Heat shock | *S. enterica* | 2.02 | 2.05 | 0.91 | 0.39 |
|  |  | IB43 | 7.37 | 0.33 | 0.58 | 0.94 |
|  |  | NRRL B-2354 | 1.93 | 2.02 | 0.92 | 0.38 |
|  | Oxidation | *S. enterica* | 0.13 | 2.24 | 0.82 | 0.41 |
|  |  | IB43 | 1.89 | 0.01 | 0.84 | 0.87 |
|  |  | NRRL B-2354 | 0.34 | 2.57 | 0.76 | 0.58 |
|  | Acid | *S. enterica* | 5.38 | 2.20 | 0.88 | 0.44 |
|  |  | IB43 | 3.51 | 0.01 | 0.98 | 0.31 |
|  |  | NRRL B-2354 | 4.80 | 2.85 | 0.70 | 0.61 |
|  | Average |  |  |  | 0.85 | 0.55 |

^a^*k*_max_, maximum inactivation rate (1/min); *N*_res_, residual population density (CFU/sample unit).

^b^RMSE, root mean square error.

^c^IB43 is the Δ*rpoS* mutant of *S.* Typhimurium ATCC 14028.

**Table S4** Parameter estimates and goodness-of-fit of log-linear with shoulder and tail for the survival of *Salmonella enterica*, *S.* Typhimurium IB43, and *Enterococcus faecium* NRRL B-2354 on raw whole almonds (RWAs) and fresh-cut leafy greens (FCLGs) during ultraviolet-C (UV-C) treatment, with or without prior exposure to sub-lethal stress.

| Food matrix | Stress | Microorganism | Parameter estimate^a^ | | | Goodness-of-fit | |
| --- | --- | --- | --- | --- | --- | --- | --- |
|  |  |  | *k*_max_ | SI | log(*N*_res_) | Adjusted *R*^2^ | RMSE^b^ |
| RWAs | No stress | *S. enterica* | 0.04 | -48.29 | 3.09 | 0.90 | 0.21 |
|  |  | IB43^c^ | 0.17 | -10.05 | 3.83 | 0.91 | 0.22 |
|  |  | NRRL B-2354 | 0.06 | -30.72 | 3.34 | 0.95 | 0.16 |
|  | Desiccation | *S. enterica* | 0.01 | -892.59 | 4.30 | 0.97 | 0.08 |
|  |  | IB43 | 0.09 | -36.25 | 2.95 | 1.00 | 0.07 |
|  |  | NRRL B-2354 | 0.20 | -0.32 | 4.94 | 0.98 | 0.04 |
|  | Heat shock | *S. enterica* | 0.01 | -1368.90 | 3.12 | 0.94 | 0.20 |
|  |  | IB43 | 0.01 | -949.03 | 2.75 | 0.95 | 0.18 |
|  |  | NRRL B-2354 | 0.57 | 7.50 | 3.70 | 0.99 | 0.08 |
|  | Oxidation | *S. enterica* | 0.07 | -85.94 | 2.24 | 0.97 | 0.19 |
|  |  | IB43 | 0.11 | -61.70 | 1.22 | 0.98 | 0.18 |
|  |  | NRRL B-2354 | 0.16 | -20.81 | 2.28 | 0.99 | 0.14 |
|  | Acid | *S. enterica* | 0.14 | -4.97 | 4.69 | 0.98 | 0.05 |
|  |  | IB43 | 0.01 | -1281.66 | 3.11 | 0.89 | 0.24 |
|  |  | NRRL B-2354 | 0.01 | -784.69 | 4.79 | 0.95 | 0.06 |
|  | Average |  |  |  |  | 0.96 | 0.14 |
| FCLGs | No stress | *S. enterica* | 0.29 | -10.07 | 0.95 | 0.99 | 0.16 |
|  |  | IB43 | 1.27 | -3.58 | -0.02 | 0.95 | 0.51 |
|  |  | NRRL B-2354 | 2.38 | 2.57 | 3.37 | 0.91 | 0.30 |
|  | Desiccation | *S. enterica* | 0.06 | -57.20 | -6.01 | 0.95 | 0.21 |
|  |  | IB43 | 1.70 | -0.01 | 0.00 | 0.63 | 1.34 |
|  |  | NRRL B-2354 | 0.07 | -58.66 | -8.51 | 0.94 | 0.29 |
|  | Heat shock | *S. enterica* | 0.36 | -12.53 | 1.78 | 0.97 | 0.26 |
|  |  | IB43 | 1.12 | -4.56 | -0.02 | 0.95 | 0.48 |
|  |  | NRRL B-2354 | 0.50 | -4.36 | 1.68 | 0.97 | 0.28 |
|  | Oxidation | *S. enterica* | 0.97 | -4.04 | 2.01 | 0.99 | 0.13 |
|  |  | IB43 | 1.74 | -3.33 | -0.05 | 1.00 | 0.09 |
|  |  | NRRL B-2354 | 1.09 | -3.13 | 2.00 | 0.98 | 0.21 |
|  | Acid | *S. enterica* | 0.30 | -20.32 | 1.89 | 0.99 | 0.12 |
|  |  | IB43 | 2.91 | -0.68 | -0.01 | 1.00 | 0.01 |
|  |  | NRRL B-2354 | 0.01 | -711.63 | -7.20 | 0.91 | 0.33 |
|  | Average |  |  |  |  | 0.94 | 0.33 |

^a^*k*_max_, maximum inactivation rate (1/min); SI, shoulder length (min); *N*_res_, residual population density (CFU/sample unit).

^b^RMSE, root mean square error.

^c^IB43 is the Δ*rpoS* mutant of *S.* Typhimurium ATCC 14028.

**Table S5** Parameter estimates and goodness-of-fit of biphasic for the survival of *Salmonella enterica*, *S.* Typhimurium IB43, *Enterococcus faecium* NRRL B-2354 on raw whole almonds (RWAs) and fresh-cut leafy greens (FCLGs) during ultraviolet-C (UV-C) treatment, with or without prior exposure to sub-lethal stress.

| Food matrix | Stress | Microorganism | Parameter estimate^a^ | | | Goodness-of-fit | |
| --- | --- | --- | --- | --- | --- | --- | --- |
|  |  |  | *f* | *k*_max1_ | *k*_max2_ | Adjusted *R*^2^ | RMSE^b^ |
| RWAs | No stress | *S. enterica* | 0.85 | 0.21 | 0.04 | 0.90 | 0.21 |
|  |  | IB43^c^ | 0.94 | 0.66 | 0.04 | 0.97 | 0.13 |
|  |  | NRRL B-2354 | 0.92 | 0.18 | 0.03 | 0.93 | 0.19 |
|  | Desiccation | *S. enterica* | 0.93 | 3.48 | 0.01 | 0.99 | 0.04 |
|  |  | IB43 | 0.96 | 0.83 | 0.06 | 0.97 | 0.18 |
|  |  | NRRL B-2354 | 0.73 | 0.24 | 0.00 | 0.99 | 0.03 |
|  | Heat shock | *S. enterica* | 0.98 | 3.57 | 0.04 | 0.94 | 0.19 |
|  |  | IB43 | 0.98 | 1.41 | 0.03 | 0.90 | 0.28 |
|  |  | NRRL B-2354 | 0.99 | 0.25 | 0.01 | 0.92 | 0.26 |
|  | Oxidation | *S. enterica* | 0.99 | 4.83 | 0.05 | 0.89 | 0.35 |
|  |  | IB43 | 0.99 | 5.31 | 0.07 | 0.92 | 0.39 |
|  |  | NRRL B-2354 | 0.99 | 0.41 | 0.05 | 0.92 | 0.35 |
|  | Acid | *S. enterica* | 0.85 | 0.19 | 0.00 | 0.98 | 0.05 |
|  |  | IB43 | 0.96 | 0.74 | 0.03 | 0.93 | 0.20 |
|  |  | NRRL B-2354 | 0.72 | 0.39 | 0.01 | 0.98 | 0.04 |
|  | Average |  |  |  |  | 0.95 | 0.19 |
| FCLGs | No stress | *S. enterica* | 0.99 | 0.42 | 0.01 | 0.94 | 0.43 |
|  |  | IB43 | 0.99 | 2.31 | 0.47 | 0.71 | 1.22 |
|  |  | NRRL B-2354 | 0.99 | 0.98 | 0.03 | 0.91 | 0.30 |
|  | Desiccation | *S. enterica* | 0.99 | 1.46 | 0.04 | 0.95 | 0.33 |
|  |  | IB43 | 0.99 | 7.45 | 0.75 | 0.92 | 0.62 |
|  |  | NRRL B-2354 | 0.99 | 0.70 | 0.02 | 0.94 | 0.39 |
|  | Heat shock | *S. enterica* | 0.99 | 2.05 | 0.00 | 0.89 | 0.43 |
|  |  | IB43 | 0.99 | 7.39 | 0.27 | 0.94 | 0.57 |
|  |  | NRRL B-2354 | 0.99 | 1.96 | 0.01 | 0.90 | 0.42 |
|  | Oxidation | *S. enterica* | 0.91 | 1.02 | 0.08 | 0.98 | 0.14 |
|  |  | IB43 | 0.99 | 2.04 | 0.38 | 0.69 | 1.23 |
|  |  | NRRL B-2354 | 0.96 | 1.00 | 0.08 | 0.95 | 0.27 |
|  | Acid | *S. enterica* | 0.99 | 5.44 | 0.03 | 0.91 | 0.39 |
|  |  | IB43 | 0.99 | 3.51 | 0.06 | 0.97 | 0.44 |
|  |  | NRRL B-2354 | 0.99 | 5.44 | 0.06 | 0.94 | 0.26 |
|  | Average |  |  |  |  | 0.90 | 0.50 |

^a^*f*, proportion of the sensitive subpopulation; *k*_max1_, inactivation rate of the sensitive subpopulation (1/min); *k*_max2_, inactivation rate of the resistant subpopulation (1/min).

^b^RMSE, root mean square error.

^c^IB43 is the Δ*rpoS* mutant of *S.* Typhimurium ATCC 14028.

**Table S6** Parameter estimates and goodness-of-fit of biphasic with shoulder for the survival of *Salmonella enterica*, *S.* Typhimurium IB43, and *Enterococcus faecium* NRRL B-2354 on raw whole almonds (RWAs) and fresh-cut leafy greens (FCLGs) during ultraviolet-C (UV-C) treatment, with or without prior exposure to sub-lethal stress.

| Food matrix | Stress | Microorganism | Parameter estimate^a^ | | | | Goodness-of-fit | |
| --- | --- | --- | --- | --- | --- | --- | --- | --- |
|  |  |  | *f* | *k*_max1_ | *k*_max2_ | SI | Adjusted *R*^2^ | RMSE^b^ |
| RWAs | No stress | *S. enterica* | 0.85 | 0.21 | 0.04 | 0.00 | 0.86 | 0.24 |
|  |  | IB43^c^ | 0.94 | 0.66 | 0.04 | 0.00 | 0.96 | 0.15 |
|  |  | NRRL B-2354 | 0.92 | 0.18 | 0.03 | 0.00 | 0.90 | 0.21 |
|  | Desiccation | *S. enterica* | 0.93 | 3.48 | 0.01 | 0.00 | 0.99 | 0.04 |
|  |  | IB43 | 0.96 | 0.83 | 0.06 | 0.00 | 0.95 | 0.21 |
|  |  | NRRL B-2354 | 0.72 | 0.30 | 0.00 | 1.65 | 0.99 | 0.03 |
|  | Heat shock | *S. enterica* | 0.98 | 3.57 | 0.04 | 0.00 | 0.92 | 0.98 |
|  |  | IB43 | 0.98 | 1.41 | 0.03 | 0.00 | 0.86 | 0.98 |
|  |  | NRRL B-2354 | 0.98 | 0.68 | 0.02 | 7.81 | 1.00 | 0.98 |
|  | Oxidation | *S. enterica* | 0.99 | 4.83 | 0.05 | 0.00 | 0.86 | 0.41 |
|  |  | IB43 | 0.99 | 5.31 | 0.07 | 0.00 | 0.89 | 0.45 |
|  |  | NRRL B-2354 | 0.99 | 0.41 | 0.05 | 0.00 | 0.89 | 0.41 |
|  | Acid | *S. enterica* | 0.85 | 0.19 | 0.00 | 0.00 | 0.97 | 0.06 |
|  |  | IB43 | 0.96 | 0.74 | 0.03 | 0.00 | 0.90 | 0.23 |
|  |  | NRRL B-2354 | 0.71 | 0.48 | 0.01 | 0.93 | 0.97 | 0.05 |
|  | Average |  |  |  |  |  | 0.93 | 0.20 |
| FCLGs | No stress | *S. enterica* | 0.99 | 0.41 | 0.00 | 0.00 | 0.92 | 0.49 |
|  |  | IB43 | 1.00 | 2.31 | 0.47 | 0.00 | N.A.^d^ | N.A. |
|  |  | NRRL B-2354 | 0.98 | 9.96 | 0.03 | 2.90 | 0.97 | 0.18 |
|  | Desiccation | *S. enterica* | 0.99 | 1.46 | 0.04 | 0.00 | 0.93 | 0.38 |
|  |  | IB43 | 1.00 | 252.16 | 0.91 | 0.00 | N.A. | N.A. |
|  |  | NRRL B-2354 | 0.99 | 1.01 | 0.04 | 0.00 | 0.92 | 0.45 |
|  | Heat shock | *S. enterica* | 0.99 | 2.05 | 0.00 | 0.00 | 0.86 | 0.99 |
|  |  | IB43 | 1.00 | 7.39 | 0.27 | 0.00 | N.A. | 100.00 |
|  |  | NRRL B-2354 | 0.99 | 1.96 | 0.01 | 0.00 | 0.87 | 0.99 |
|  | Oxidation | *S. enterica* | 0.91 | 1.02 | 0.08 | 0.00 | 0.97 | 0.16 |
|  |  | IB43 | 1.00 | 2.04 | 0.38 | 0.00 | N.A. | N.A. |
|  |  | NRRL B-2354 | 0.96 | 1.00 | 0.08 | 0.00 | 0.93 | 0.31 |
|  | Acid | *S. enterica* | 0.99 | 5.44 | 0.03 | 0.00 | 0.88 | 0.45 |
|  |  | IB43 | 1.00 | 3.59 | 0.00 | 0.00 | N.A. | N.A. |
|  |  | NRRL B-2354 | 0.99 | 7.22 | 0.06 | 0.00 | 0.92 | 0.30 |
|  | Average |  |  |  |  |  | 0.92 | 0.37 |

^a^*f*, proportion of the sensitive subpopulation; *k*_max1_, inactivation rate of the sensitive subpopulation (1/min); *k*_max2_, inactivation rate of the resistant subpopulation (1/min); SI, shoulder length (min).

^b^RMSE, root mean square error.

^c^IB43 is the Δ*rpoS* mutant of *S.* Typhimurium ATCC 14028.

^d^The parameter estimate for *f* is exactly one, indicating that the model is not applicable to the observed data.

# Supplementary figures


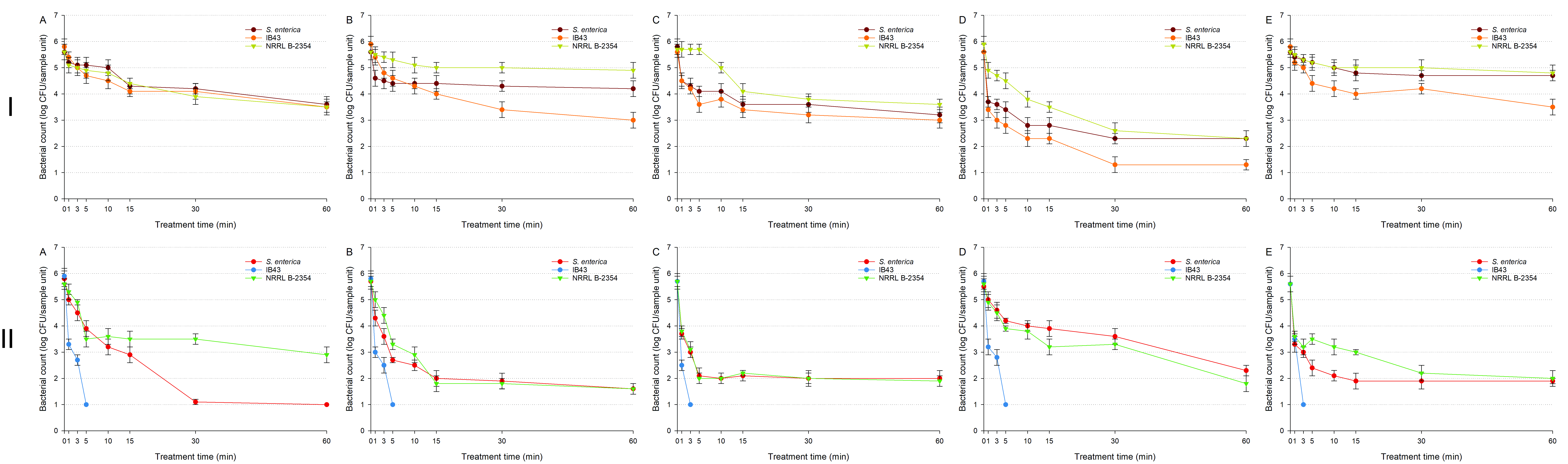


**Figure S1** Survival of *Salmonella enterica*, *S.* Typhimurium IB43 (Δ*rpoS* mutant of *S.* Typhimurium ATCC 14028 wild-type), and *Enterococcus faecium* NRRL B-2354 on raw whole almonds (I) and fresh-cut leafy greens (II) during ultraviolet-C (UV-C) treatment, with or without prior exposure to sub-lethal stress. Bacterial survival is shown for unstressed cells (A) and cells subjected to sub-lethal desiccation (B), heat shock (C), oxidation (D), or acid (E) stress. UV-C treatment was applied for 30 (I) or 60 min (II). Bacterial counts are not shown at certain time points because no cells were detected, even after enrichment. Error bars represent standard deviations from three independent trials. Bacterial counts plotted as 1.0 log CFU/sample unit without error bars were below the limit of detection by direct plating but were detectable by enrichment.


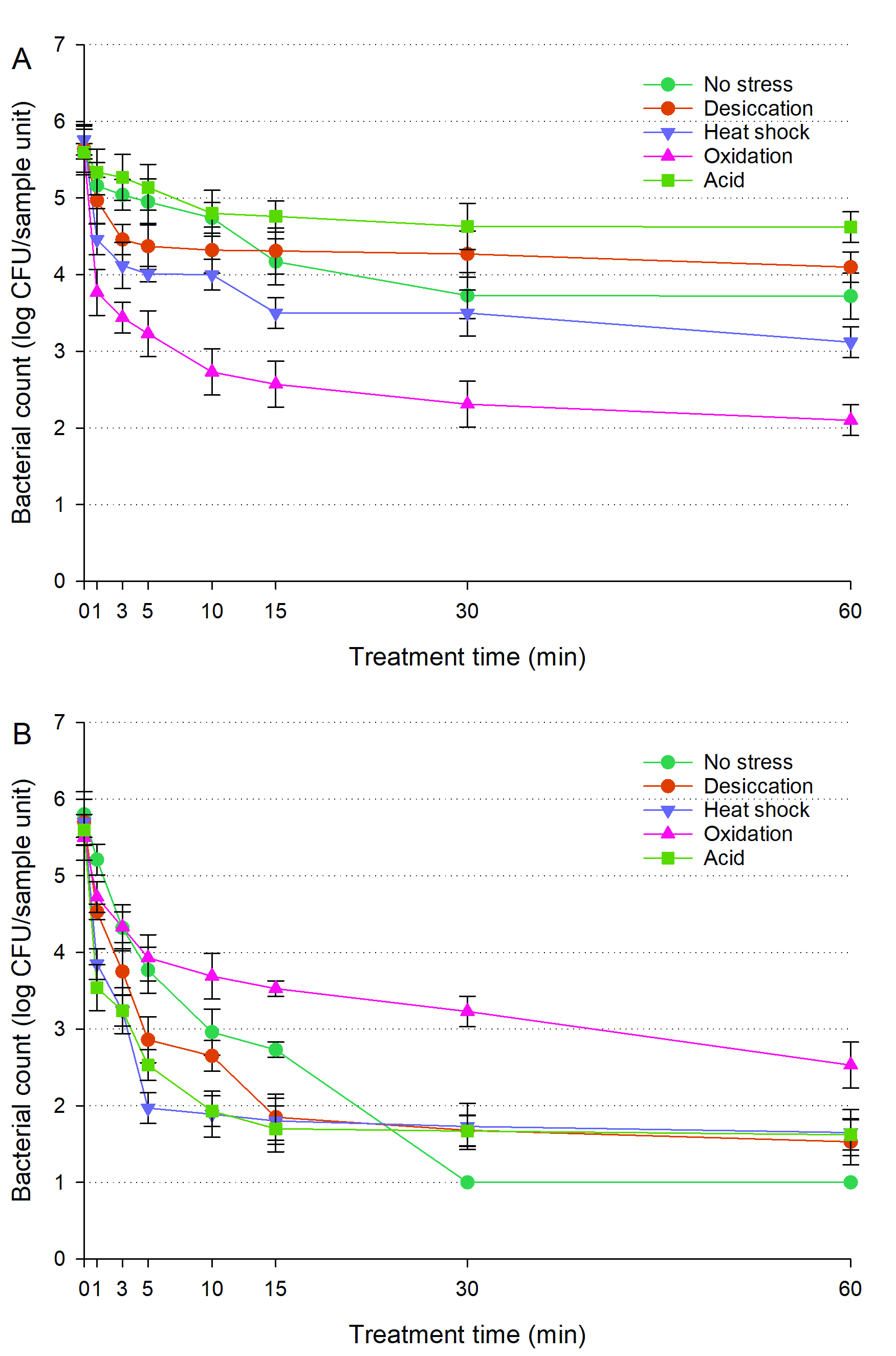


**Figure S2** Survival of *Salmonella enterica* Typhimurium ATCC 14028 wild-type on raw whole almonds (A) and fresh-cut leafy greens (B) during ultraviolet-C treatment, with or without prior exposure to sub-lethal stress. Error bars represent standard deviations from three independent trials. Bacterial counts plotted as 1.0 log CFU/sample unit without error bars were below the limit of detection by direct plating but were detectable by enrichment.


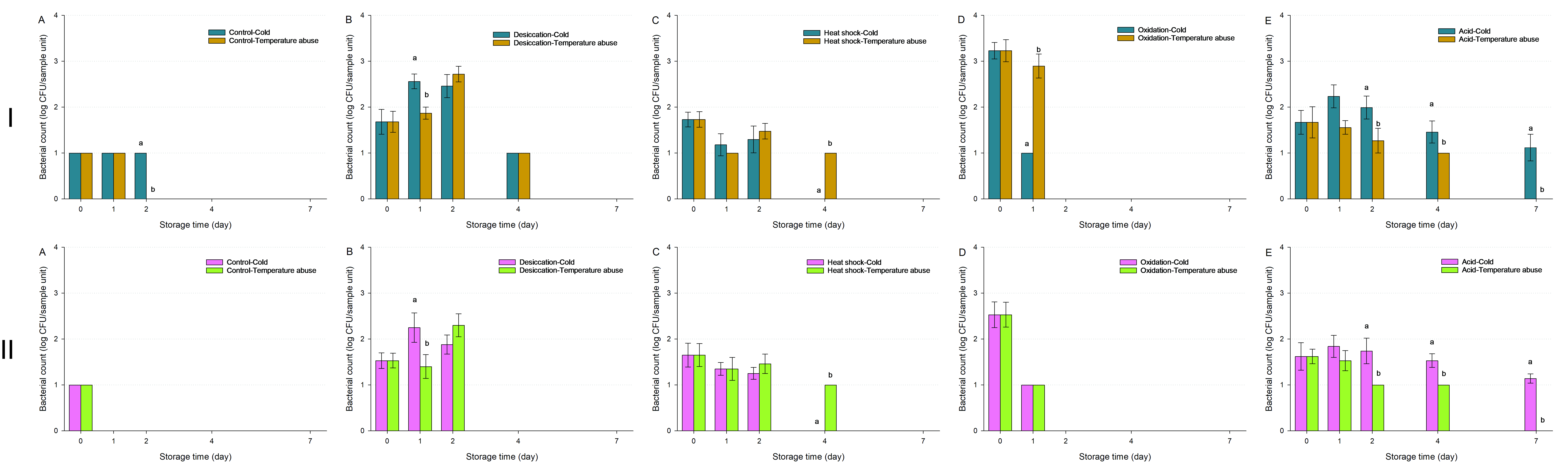


**Figure S3** Survival of *Salmonella enterica* Typhimurium ATCC 14028 wild-type on fresh-cut leafy greens during a seven-day storage period under cold or temperature abuse condition following ultraviolet-C (UV-C) treatment. Bacterial survival is shown for unstressed cells (A) and cells subjected to sub-lethal desiccation (B), heat shock (C), oxidation (D), or acid (E) stress. UV-C treatment was applied for 30 (I) or 60 min (II). Bacterial counts are not shown at certain time points because no cells were detected, even after enrichment. Error bars represent standard deviations from three independent trials. Bacterial counts plotted as 1.0 log CFU/sample unit without error bars were below the limit of detection by direct plating but were detectable by enrichment. Bacterial counts are not shown at certain time points because no cells were detected, even after enrichment. Different letters above bars indicate significant differences (*p* < 0.05) between cold and temperature abuse conditions.
